# Intranasal respiratory syncytial virus vaccine attenuated by codon-pair deoptimization of seven open reading frames is genetically stable and elicits mucosal and systemic immunity and protection against challenge virus replication in hamsters

**DOI:** 10.1371/journal.ppat.1012198

**Published:** 2024-05-13

**Authors:** Megan Levy, Jessica W. Chen, Jaclyn A. Kaiser, Hong-Su Park, Xueqiao Liu, Lijuan Yang, Celia Santos, Ursula J. Buchholz, Cyril Le Nouën

**Affiliations:** RNA Viruses Section, Laboratory of Infectious Diseases, NIAID, NIH, Bethesda, Maryland, United States of America; NYU School of Medicine, UNITED STATES

## Abstract

Respiratory syncytial virus (RSV) is the most important viral agent of severe pediatric respiratory illness worldwide, but there is no approved pediatric vaccine. Here, we describe the development of the live-attenuated RSV vaccine candidate Min AL as well as engineered derivatives. Min AL was attenuated by codon-pair deoptimization (CPD) of seven of the 11 RSV open reading frames (ORFs) (NS1, NS2, N, P, M, SH and L; 2,073 silent nucleotide substitutions in total). Min AL replicated efficiently *in vitro* at the permissive temperature of 32°C but was highly temperature sensitive (shut-off temperature of 36°C). When serially passaged at increasing temperatures, Min AL retained greater temperature sensitivity compared to previous candidates with fewer CPD ORFs. However, whole-genome deep-sequencing of passaged Min AL revealed mutations throughout its genome, most commonly missense mutations in the polymerase cofactor P and anti-termination transcription factor M2-1 (the latter was not CPD). Reintroduction of selected mutations into Min AL partially rescued its replication *in vitro* at temperatures up to 40°C, confirming their compensatory effect. These mutations restored the accumulation of positive-sense RNAs to wild-type (wt) RSV levels, suggesting increased activity by the viral transcriptase, whereas viral protein expression, RNA replication, and virus production were only partly rescued. In hamsters, Min AL and derivatives remained highly restricted in replication in the upper and lower airways, but induced serum IgG and IgA responses to the prefusion form of F (pre F) that were comparable to those induced by wt RSV, as well as robust mucosal and systemic IgG and IgA responses against RSV G. Min AL and derivatives were fully protective against challenge virus replication. The derivatives had increased genetic stability compared to Min AL. Thus, Min AL and derivatives with selected mutations are stable, attenuated, yet highly-immunogenic RSV vaccine candidates that are available for further evaluation.

## Author summary

Synonymous recoding of codon-pair usage is an emerging strategy for expedited development of live-attenuated vaccines. Respiratory syncytial virus (RSV) is the most important viral agent of severe respiratory illness in children worldwide, but a pediatric vaccine for active immunization is not yet available. Here, we developed a new genome-scale codon-pair deoptimized (CPD) RSV vaccine candidate, namely Min AL, containing 2,073 silent nucleotide mutations spread over seven different ORFs. Min AL replicated efficiently at the permissive temperature of 32°C but was highly temperature sensitive. When serially passaged at increasing temperatures, Min AL remained highly temperature sensitive at 39–40°C. However, sequence analysis showed that it had acquired numerous missense mutations in several CPD and non-CPD genes. Reintroduction of several of these mutations into Min AL decreased its temperature sensitivity, rescued viral transcription to wt RSV levels, partially increased RNA replication, protein expression, and virus replication, albeit not to wt levels, and increased genetic stability. In hamsters, intranasal immunization with Min AL and derivatives induced robust mucosal and systemic anti-RSV antibody responses and full protection against challenge virus replication. This study identified Min AL and derivatives as highly attenuated, yet highly immunogenic and genetically stabilized CPD RSV vaccine candidates appropriate for further evaluation.

## Introduction

Codon-pair deoptimization (CPD) is a strategy for synonymous recoding of one or more open reading frames (ORFs) of a microbial pathogen (usually a virus) in order to create new versions of the pathogen with reduced fitness [1–3]. CPD arises from the previously-described phenomenon of codon-pair bias, in which certain pairs of synonymous codons are overrepresented in the wild-type (wt) pathogen (and its host) compared to others [4]. CPD involves rearranging codons by computer algorithm to increase the proportion of codon pairs that normally are under-represented, without changing the overall frequency of individual codons or the amino acid (aa) coding [1]. This provides—in an expedited manner—attenuated versions of viral pathogens as new vaccine candidates [1].

The attenuation that is introduced by CPD might involve a number of factors, including a reduction in translation efficiency, an increase in toll-like receptor signaling due to the increased numbers of immunostimulatory CpG and UpA dinucleotides, and a reduction in mRNA stability and/or synthesis [5–8]. CPD can involve hundreds or thousands of nucleotide (nt) substitutions, which might provide stable attenuation phenotypes. Another advantage of CPD is that the level of attenuation can theoretically be modulated by increasing or decreasing the number of CPD ORFs in a recoded virus. Importantly, CPD viruses frequently have proved to be as immunogenic as their wt counterparts despite being highly attenuated [3]. The CPD strategy has provided live-attenuated viral vaccine candidates against clinically-important RNA viruses such as human immunodeficiency virus type-1, Influenza, SARS-CoV-2, poliovirus or Zika virus [1,9–12] as well as DNA viruses, including Marek’s disease virus, an alphaherpesvirus type 2 that is a major poultry pathogen [13]. Some of the human vaccine candidates are being evaluated in Phase I clinical trials (e.g., Clinicaltrials.gov study ID NCT05223179, influenza virus H1N1; NCT04919109, RSV) and a Phase III clinical trial (ISRCNT15779782, SARS-CoV-2).

Respiratory syncytial virus (RSV) belongs to the order Mononegavirales, family *Pneumoviridae*. It is an enveloped virus with a negative-sense, non-segmented, single-stranded RNA genome of approximately 15.2 kilobases. Its genome is transcribed by the viral polymerase into 10 mRNAs encoding 11 proteins, with the M2 mRNA encoding two proteins, M2-1 (transcription processivity protein) and M2-2 (protein involved in regulating transcription and RNA replication), from two overlapping ORFs. Each gene is flanked with a pair of gene-start and gene-end transcription signal sequences. RSV is the most important viral agent of severe respiratory illness in infants and young children [14–16].

In 2023, two passive-immunoprophylaxis approaches were approved for use in the US to protect young infants against RSV; a maternal RSV vaccine based on the pre-fusion form of the RSV F surface glycoprotein [17] and a long-lasting monoclonal antibody for infants under 8 months of age [18,19]. However, protection by these passive approaches will wane during the first year of life and there are currently no vaccines for active immunization to reduce the substantial burden of severe RSV illness in older infants and young children. Live-attenuated vaccines for intranasal (IN) immunization are considered safe for infants as they are not associated with a risk of priming for vaccine-enhanced RSV disease [20–27], unlike killed and subunit RSV vaccines [28–31]. Furthermore, when administered intranasally, live-attenuated vaccines can induce immunity directly at the site of viral entry, disease, and spread, and also induce systemic immunity. They are also particularly efficient to induce antigen-specific tissue-resident memory T cells that might help to provide long-term protection [32].

**Fig 1:**
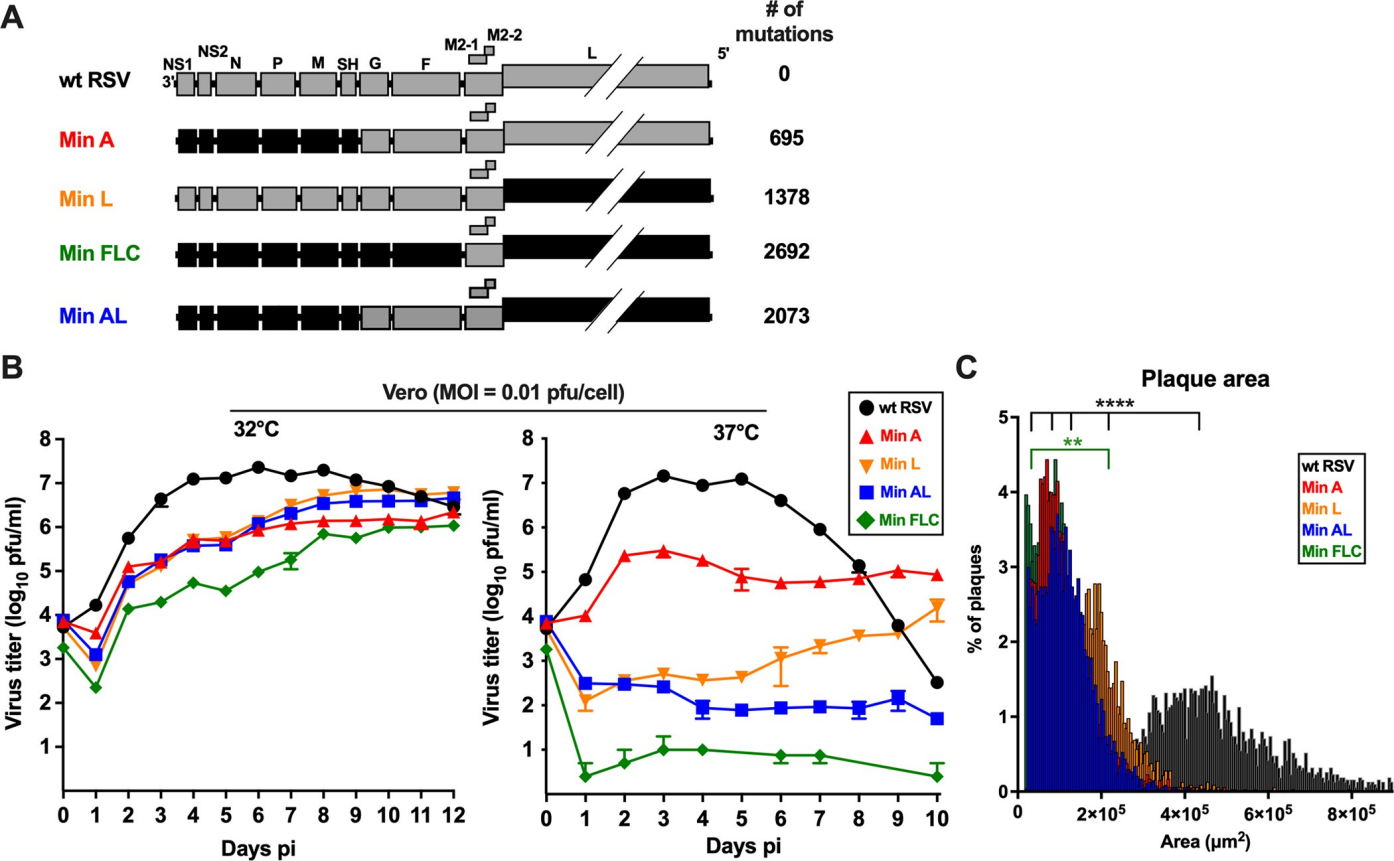
Construction of Min AL and characterization *in vitro*. **A.** Genome map of the newly constructed Min AL virus compared to wt RSV and the previously-described Min A, Min L and Min FLC viruses [33]. CPD and wt ORFs are shown in black and grey, respectively. The number of silent nucleotide substitutions introduced by CPD in each construct is indicated to the right. **B.** Multi-cycle replication kinetics in Vero cells. Cell monolayers in six-well plates were infected using an MOI of 0.01 pfu/cell with the indicated viruses and incubated at 32°C (left panel) or 37°C (right panel). At 24 h intervals, cells from duplicate wells for each virus were scraped into the medium, vortexed to release cell-associated virus, the suspensions were clarified by low-speed centrifugation, and the media supernatants were harvested, aliquoted, and snap-frozen. Virus titers were determined later by immunoplaque assay at 32°C. Titers correspond to the mean of two replicate titrations of virus from each of two replicate wells at each timepoint. Day 0 titers correspond to the back-titration of the inocula. **C.** Plaque sizes. Vero cells in six-well plates were infected with 250 pfu/well of the indicated virus and incubated under methylcellulose at 32°C. At day seven pi, plates were fixed and stained with a mixture of three anti-RSV F MAbs and a PE-labeled secondary antibody. The plaque area (in μm^2^) was evaluated on an average of 3451 (±1200) plaques per virus (** = p≤0.01, **** = p≤0.0001, Wilcoxon rank test with continuity correction post hoc test).

We previously generated four CPD RSV vaccine candidates (Min A, Min B, Min L and Min FLC) that contained various combinations of one or more CPD ORFs [33] (Min A, Min L, and Min FLC are shown in Fig 1A). These viruses were temperature sensitive and exhibited a range of attenuation *in vitro* and *in vivo* [33]. While Min B (CPD G and F) and Min FLC (all ORFs CPD except M2-1 and M2-2; Fig 1A) were deemed over-attenuated *in vitro* and *in vivo*, Min A (CPD NS1, NS2, N, P, M and SH; Fig 1A) and Min L (CPD L; Fig 1A) were evaluated further [33,34]. In these previous studies, the genetic stability of Min A and Min L was evaluated by serial passage *in vitro* at increasing temperatures. Subsequent whole-genome deep sequencing of selected passages identified numerous prominent mutations in various CPD and non-CPD genes, most notably in the N-terminal domain of polymerase cofactor P in Min A and between aa 70–90 of the anti-termination transcription factor M2-1 in Min L [35,36]. Reintroduction of these mutations into the respective CPD parent genomes partially rescued replication of Min A and Min L at increasing temperatures. This resulted in CPD RSV viruses with increased genetic stability and—somewhat paradoxically—increased attenuation and immunogenicity in the hamster model [35,36]. A stabilized version of Min L containing four mutations reintroduced into the N, P, M2-1 and L genes (NPM2-1[N88K]L virus) was safe and immunogenic in non-human primates [37], and safe in a phase I clinical study in elderly (Clinicaltrials.gov, NCT04295070). This candidate is currently being evaluated in RSV-seropositive and -seronegative children between 6 months and 5 years of age (Clinicaltrials.gov, NCT04919109). However, results from this Phase I study will not be available soon and data will be limited to a relatively small number of children. While this study is ongoing, it would be prudent to generate and characterize alternative candidates, preferably ones based on additional attenuating elements with improved phenotypes. If the outcome of ongoing Phase I studies suggests that changes in the tested vaccines would be desirable, appropriate backup candidates would immediately be available.

Thus, in the present study, we designed, rescued, and evaluated Min AL, a new CPD RSV that combines the CPD NS1, NS2, N, P, M, and SH ORFs of Min A [35] with the CPD L ORF of Min L [36]. Increasing the number of synonymous mutations in Min AL by combining the CPD ORFs of Min A and Min L has the potential to increase attenuation and increase genetic and phenotypic stability compared to Min A and Min L. The increased number of CpG and UpA in Min AL compared to Min A and Min L also has the potential to increase immunogenicity. Passage of Min AL *in vitro* with incremental increases in temperature identified a broad array of potentially-compensatory mutations, most notably in P and M2-1. Inclusion of some of these mutations into Min AL provided increased genetic and phenotypic stability and immunogenicity. The present study describes the evaluation of Min AL and several derivatives *in vitro* and in the hamster model, and identifies improved vaccine candidates for further development.

## Results

### Construction and rescue of Min AL

The new CPD virus Min AL was created by combining the CPD ORFs of Min A (CPD NS1, NS2, N, P, M, SH) and Min L (CPD L) (Fig 1A). Thus, Min AL contains CPD versions of seven of the 11 RSV ORFs, with a total of 2,073 silent mutations within ORFs. This represents a 3- and 1.5-fold increase over Min A (695 silent mutations) and Min L (1,378 silent mutations; Fig 1A), respectively. The full-length genome of wt RSV contained only 87 CpGs and 1,462 UpAs, while that of Min AL contained 426 CpGs and 1,770 UpAs. When the analysis was focused on the combined ORFs, wt RSV contains 79 CpGs and 1,312 UpAs, Min A contains 205 CpGs and 1,418 UpAs, Min L contains 292 CpGs and 1,514 UpAs, and Min AL contains 418 CpGs and 1,620 UpAs (S1 Table). Min AL was readily rescued by reverse genetics as previously described [33] and passaged once to prepare working stocks. Illumina whole-genome deep-sequencing of Min AL stocks confirmed that the recovered genomes were free of any adventitious mutations with an abundance of ≥1% of reads.

### Min AL combines temperature sensitivity from Min A and Min L

We first evaluated the replication of Min AL in comparison to the previous CPD viruses in multi-cycle *in vitro* growth kinetics experiments (Fig 1B). Vero cells were infected with Min A, Min L, Min AL, Min FLC or wt RSV using an multiplicity of infection (MOI) of 0.01 plaque forming units (pfu)/cell. Infected cells were incubated at 32°C, a permissive temperature for temperature-sensitive mutants that also simulates the temperature of the upper respiratory tract, or the more-restrictive temperature of 37°C that simulates the temperature of the lower respiratory tract and core body. Virus replication was evaluated for 12 and 10 days, respectively.

At 32°C (Fig 1B, left panel), wt RSV replicated efficiently, reaching 7.4 log_10_ pfu/ml by day 6 post-infection (pi), as is typically observed. Replication of all CPD RSVs was reduced and delayed, with replication of Min FLC being the most affected, reaching a maximum peak titer of 6.0 log_10_ pfu/ml by day 12 pi, similar to our previous results [33]. Replication of the new Min AL virus was comparable to Min L, and slightly more efficient than Min A, reaching a peak titer of 6.7 log_10_ pfu/ml on day 12 pi. At 37°C (Fig 1B, right panel), wt RSV replicated efficiently and reached a peak titer on day 3 pi (7.2 log_10_ pfu/ml). As previously described [33], replication of Min A and Min L was strongly reduced (by 48-fold and 1000-fold, respectively) and only a baseline replication of Min FLC was detected. The replication of Min AL was reduced compared to Min L by about 60-fold, indicating that the new virus was more temperature sensitive than Min A and Min L.

The plaque sizes of wt and CPD RSVs at 32°C on Vero cells were evaluated following incubation for seven days under a methylcellulose overlay (Fig 1C). Compared to wt RSV, the plaque sizes of all CPD RSVs were significantly reduced by 3- to 5-fold (p<0.0001). The plaques formed by Min AL appeared to be slightly smaller than Min L, slightly larger than Min A, and even larger than Min FLC, but the differences were not significant.

We next determined the shut off temperature (T_SH_) for plaque formation by Min AL, defined as the lowest temperature at which there is a reduction in plaque number compared to 32°C that is 100-fold or greater than that observed for wt RSV between the two temperatures (S2 Table). Wt RSV efficiently formed plaques at 40°C as typically observed, while the T_SH_ of Min A, Min L and Min FLC were 40°C, 38°C and 36°C, respectively, as previously described [33]. The T_SH_ of Min AL was found to be 36°C, suggesting that its temperature sensitivity phenotype had contributions from both Min A and Min L.

### Genetic and phenotypic stability of Min AL when serially passaged at increasing temperature (*in vitro* stress test)

**Fig 2:**
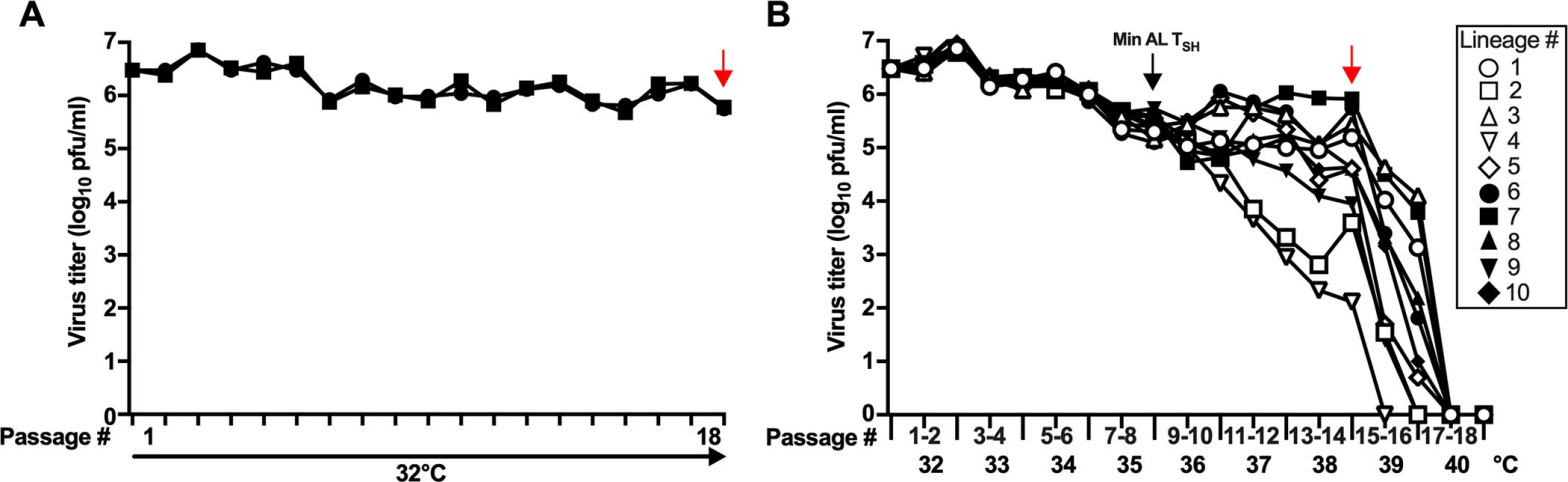
*In vitro* stress test of Min AL. Twelve replicate monolayer cultures of Vero cells in 25 cm^2^ flasks were inoculated at an MOI of 0.1 pfu/cell with Min AL and serially passaged in parallel. Each flask represented a separate lineage. Two lineages (A) were passaged 18 times at the permissive temperature of 32°C as controls. For the other ten lineages (B), the temperature of incubation was increased by one°C after every other passage from 32°C to 40°C for a total of 18 passages. When extensive syncytia were observed or when cells started to detach (typically between six and 11 days pi), cells were scraped into the medium and the supernatants were harvested and clarified by low-speed centrifugation. One ml of the total of five clarified ml was used to inoculate the following passage. The remaining supernatants were aliquoted and snap frozen in dry ice for subsequent titration at 32°C and sequence analysis. The passage number and corresponding temperatures of incubation are indicated, as well as the virus titer of the initial inoculum and passage harvests. Each lineage is represented separately by a different symbol. The passages incubated at the 36°C shut-off temperature of Min AL (T_SH_, P9 and 10) are indicated with a black arrow. Red arrows mark supernatants collected after 14 passages at increasing temperatures or 18 passages at 32°C for the controls that were used for viral RNA extraction and sequence analysis.

Since Min AL exhibited a strong temperature sensitivity, we evaluated the genetic stability and the stability of the temperature-sensitivity phenotype of Min AL in an *in vitro* stress test (Fig 2). Twelve 25 cm^2^ flasks of Vero cells were inoculated with Min AL using an initial MOI of 0.1 pfu/cell. Each flask represented an independent lineage. Ten lineages were passaged in parallel starting at the permissive temperature of 32°C, with an increase in temperature of 1°C following every second passage up to 40°C, for a total of 18 passages representing approximately 4 months of continuous culture. In parallel, two additional lineages were passaged 18 times at 32°C as controls. For each passage, cell monolayers from each lineage were harvested when the cytopathic effect was extensive, typically between 4 and 7 days after infection, and one-fifth of the clarified culture medium was used to infect a fresh monolayer of Vero cells. The remainder of the clarified medium was snap frozen in aliquots on dry ice for subsequent titration and, for supernatants collected after 14 passages at increasing temperatures or after 18 passages for the controls, for whole-genome Illumina deep sequencing.

For the two control lineages of Min AL passaged at 32°C, the virus replicated efficiently for all 18 passages, reaching titers that varied between 5.5 and 7.0 log_10_ pfu/ml (Fig 2A). Virus titers of each of the ten Min AL lineages passaged at increasing temperatures increased by 3-fold during the first two passages at 32°C, reaching on average 6.9 log_10_ pfu/ml, similar to the control lineages (Fig 2B). From P3 to P8 (33°C to 35°C), titers in each lineage decreased steadily by about 30-fold on average to 5.5 log_10_ pfu/ml. Then, from P9 to P14 (first passage at 36°C to second passage at 38°C), replication was variable among the lineages. Titers in six of the 10 lineages continued to decrease between 6- and 1,800-fold between P9 to P14; the titers in one lineage remained unchanged from P9 to P14; and titers in two lineages increased by 2- to 4-fold from P9 to P14. From P15 to P16 (the two passages at 39°C), titers sharply decreased in all lineages, ranging from 4.1 log_10_ pfu/ml to below the limit of detection. Finally, at P17 and P18 (the two passages at 40°C), replication of all Min AL lineages was below the limit of detection. The observation that most of the lineages replicated efficiently beyond the T_SH_ for Min AL of 36°C, but that none of them replicated at 40°C, suggested that they had a partial loss of the ts phenotype.

### Min AL lineages acquired multiple, different point mutations in both CPD and wt ORFs during the *in vitro* stress test

To identify mutations that might be responsible for the partial loss of the ts phenotype during the *in vitro* stress test, viral RNA was extracted from clarified cell culture supernatants harvested at the end of P14 (second passage at 38°C), corresponding to the last passage at which replication was still detected in most of the lineages (Fig 2B, red arrow). Viral RNA also was extracted from supernatants from the two control lineages at P18. The viral RNAs were amplified by overlapping RT-PCRs using RSV-specific primers. PCR amplicons covering the whole genome, with the exception of the outer-most primers at the 3’- and 5’-ends (nucleotides 1–23 and 15,174–15,223), were sequenced by Illumina deep sequencing. PCR amplicons were not obtained for P14 of lineages #2 and #4, presumably due to low viral titers, and thus these two lineages were not evaluated further.

Sequence analysis of these eight “stressed” lineages (#1, 3, 5, 6, 7, 8, 9 and 10) identified a total of 38 different prominent (>30% of the sequencing reads) point mutations (Table 1). Of these 38 different mutations, three were present in two lineages each and one in three lineages; the remaining 34 mutations were present in only one lineage each. Each lineage had 3–8 mutations with >30% abundance.

**Table 1:** Mutations detected at a frequency of >30% in eight of the ten Min AL lineages at the end of passage 14 (2^nd^ passage at 38°C) as well as in each of the two controls at passage 18 (32°C).

			Lineage no.*									
			1	3	5	6	7	8	9	10	Ct1	Ct2
Titer (log_10_ pfu/ml)			5.2	5.4	4.6	5.7	5.9	4.6	3.9	4.6	5.8	5.8
**Gene**	**nt mutation**	**aa mutation**										
NS1	t111a	S5T					95					
NS1	a236g	I46M	84									
NS1	t389a	H97Q						59		37		
NS1	a427g	E110G				32						
N	a1288t	M50L					94					
N	a1297g^†^	I53V							37			
N	a1547g^†^	K136R				34						
N	a1980g	E280E (silent)				32						
P gene start	c2334a	/	85						35			
P gene start	a2337t	/									46	
P 5’ UTR	a2344g	/								36		
P	a2402t	K19I						57				
P	g2423a^†^	G26D		92		31				51		
P	a2425c^†^	K27Q				44						
P	a2440c	K32Q			85							
P	a3057t	S237S (silent)								49		
G	c5282t	T198T (silent)			76							
F	t6934a	S425T		37								
M2-1	a7647g	H14R		37								
M2-1	g7756a	M50I		48								
M2-1	a7811g	T69A				43						
M2-1	a7816t	E70D					94					
M2-1	g7823t	A73S								41		
M2-1	t7825c	A73A (silent)						55				
M2-1	t7833c	V76A						56				
M2-1	t7866a	I87K	84									
M2-1	a7869c	N88T		41					68			
M2-1	a7869g	N88S								46		
M2-1	t7875c	I90T				45						
M2-1	a7876t	I90I (silent)						55				
M2-1	t7879c	T91T (silent)						56				
M2-1	g7898t	A98S			74							
M2-1/M2-2	c8170t	A188A (silent)/ P4L		54								
L gene start	a8494g	/		67								
L	t9011c	L171L (silent)					94					
L	t10781c^†^^‡^^§^	N761N (silent)			82							
L	t12245a^†^	N1249K					95					
L	t13028c^†^	S1510S (silent)		63								
L	c14726a^†^^‡^	S2076S (silent)							36			

“/” indicates that the amino acid mutation is not applicable for this particular mutation as the given mutation is localized in a non-translated region.

*Percentage of reads with the indicated mutation; only mutations present in >30% of the reads are shown.

Missense mutations and mutations in non-translated regions present in >50% of the reads are highlighted in yellow.

Nucleotide numbering is based on RSV sequence KT992094.

^†^Mutations involving a codon that had been changed as part of CPD of NS1, NS2, N, P, M, SH, or L

^‡^Mutations involving a nucleotide that had been changed as part of CPD of NS1, NS2, N, P, M, SH or L.

^§^Mutation involving a nucleotide that had been changed as part of CPD of NS1, NS2, N, P, M, SH or L and that restored wt sequence.

Of the 38 prominent mutations from the stressed lineages, only three were in non-protein-coding regions: namely, in the P gene-start signal (c2334a, the 5^th^ nucleotide of the signal GGGGCAAAT, found in two lineages), the 5’ untranslated region (UTR) of the P gene (a2344g), and the L gene-start signal (a8494g, the 4^th^ nucleotide of the signal GGGACAAAAT). The remaining 35 mutations (92%) were in ORFs; of these, 25 (71%) were missense mutations, suggesting a selective pressure for aa change. Of these 25 missense mutations, four (16%) were in NS1, three (12%) in N, four (16%) in P, one (4%) in F, 11 (44%) in M2-1, one (4%) in M2-2 and one (4%) in L. Thus, Min AL lineages passaged under increasing temperature acquired missense mutations in the CPD NS1, N, P and L ORFs but also in the F, M2-1 and M2-2 ORFs which had not been subjected to CPD, with about half of the missense mutations found in M2-1.

After 18 passages of the two control lineages at the permissive temperature of 32°C, only a single point mutation of >30% abundance was identified, occurring in one of the two lineages (Table 1, Ct1). This mutation was located in the P gene-start signal (a2337t at the 8^th^ nucleotide of the signal GGGGCAAAT). Thus, Min AL was genetically stable at the permissive temperature.

### Reintroduction of mutations identified at P14 of the *in vitro* stress test (second passage at 38°C) into the Min AL backbone

We next attempted to identify the mutations responsible for the partial loss of the ts phenotype of Min AL during the *in vitro* stress test (Table 1). To do so, we considered four lineages (#1, 3, 6 and 7, Table 1) that replicated the most efficiently at P14 (titers >5.0 log_10_ pfu/ml) and thus might be expected to contain mutations with the strongest compensatory effects on the ts phenotype and virus replication. Of these four lineages, lineage #6 contained six missense mutations, with none present at >45% of the reads, suggesting that this lineage contained mixed virus populations that might be difficult to study. Thus, this lineage was not evaluated further. Lineage #1 contained three prominent mutations, each present in >50% of the reads, in the NS1 ORF (I46M), the P gene-start signal (c2334a), and the M2-1 ORF (I87K). Lineage #3 contained eight prominent mutations, three of which were of >50% abundance: namely, one each in the P ORF (G26D), the M2-2 ORF (P4L), and the L gene-start signal (a8494g). Lineage #7 contained five prominent mutations, all with >90% abundance. Four of these were missense mutations in the NS1 ORF (S5T), the N ORF (M50L), the M2-1 ORF (E70D), and the L ORF (N1249K), and the other was a silent mutation in the L ORF.

**Fig 3:**
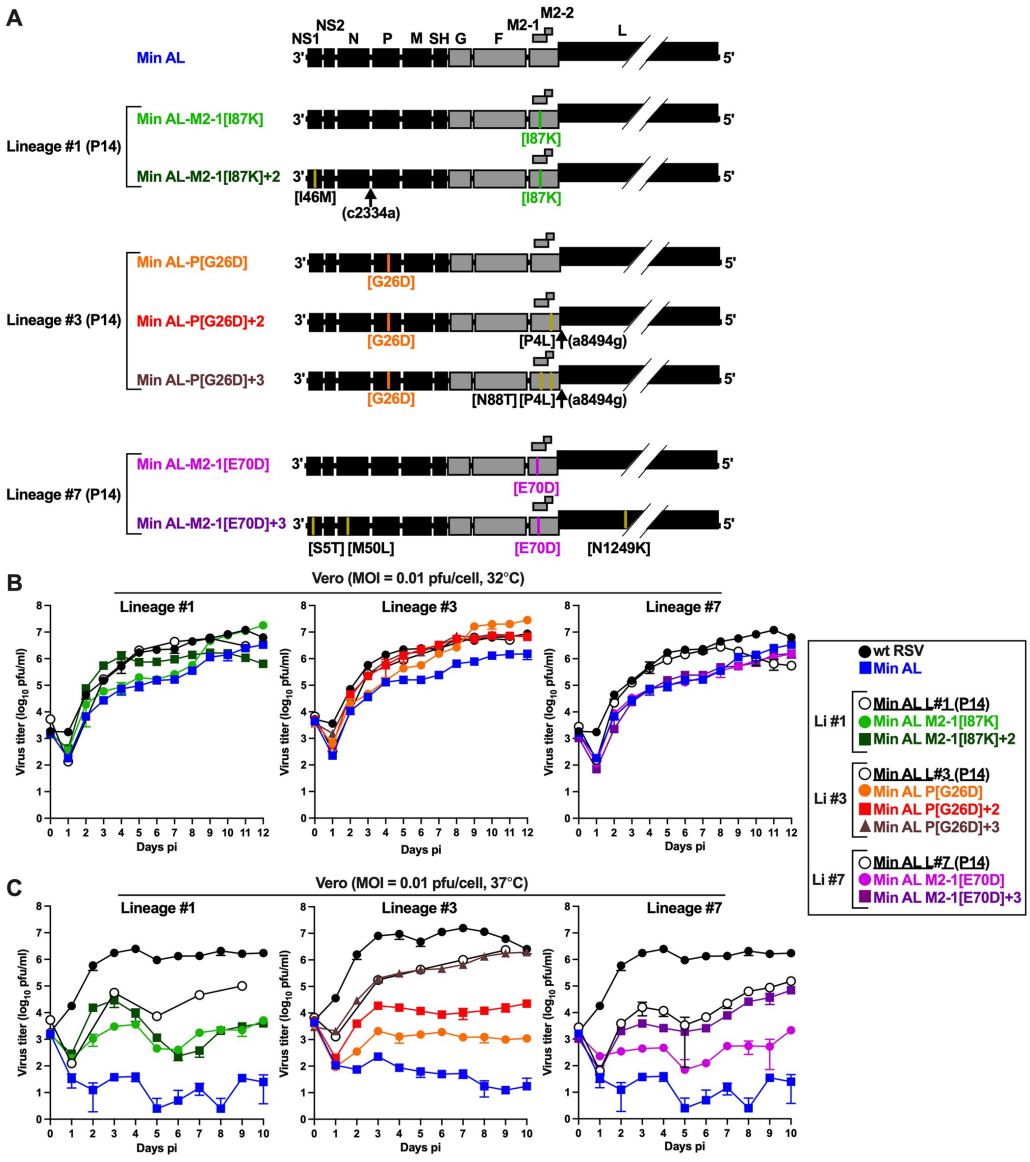
Prominent mutations identified from the *in vitro* stress test incrementally reduce the temperature sensitivity of Min A. **A.** Genome maps of Min AL and derivatives containing the reintroduction of one or more prominent (>50% abundance) mutations identified by whole-genome deep sequencing of passage 14 (P14) of lineages #1, 3 and 7 (Table 1). Reintroduced mutations are identified underneath the genome of each Min AL derivative and are described in the Results. The two viruses at the top contain the indicated mutations from lineage #1; the next three viruses contain the indicated mutations from lineage #3, and the bottom two viruses contain the indicated mutations from lineage #7. **B-C.** Multi-cycle replication kinetics of Min AL and derivatives. Replicate Vero monolayers in six-well plates were infected using an of MOI of 0.01 pfu/cell with wt RSV, Min AL, P14 supernatant from lineages #1, 3 or 7 of the *in vitro* stress test, or Min AL derivatives containing the indicated reintroduced mutations, and incubated at 32°C (B) or 37°C (C). Titers for Min AL, its derivatives, and wt RSV are the means with standard deviation of two replicate titrations each of two replicate wells, harvested at each timepoint as described in the legend of Fig 1C. Due to limited sample availability, titers of the P14 lineages #1 and #3 are the means of two replicate titrations of one well at every other time point. Day 0 titers are the back titration of the inocula. Min AL derivatives containing mutations from lineages #1 and #7 (B, C; right and left panels) were evaluated in a first experiment, and Min AL derivatives containing mutations from lineage #3 (B, C; middle panels) were evaluated in a second independent experiment. Wt RSV and Min AL were included in both experiments.

Based on these observations, two derivatives of Min AL were made using mutations from lineage #1 (Fig 3A, top): one derivative contained the mutation M2-1[I87K] alone (virus Min AL-M2-1[I87K]) and a second derivative contained this mutation plus two additional mutations, namely NS1[I46M] and the P gene-start signal mutation (c2334a) (virus Min AL-M2[I87K]+2).

Three additional derivatives of Min AL were made using mutations from lineage #3 (Fig 3A, middle): one derivative contained the mutation P[G26D] alone (virus Min AL-P[G26D]); a second derivative contained this mutation plus two additional mutations, namely M2-2[P4L] and the L gene-start signal mutation (a8494g) (virus Min AL-P[G26D]+2); and a third derivative contained these three mutations combined with the M2-1[N88T] mutation (Min AL-P[G26D]+3).

Two additional derivatives of Min AL were made using mutations from lineage #7 (Fig 3A, bottom): one derivative contained the mutation M2-1[E70D] alone (virus Min AL-M2-1[E70D]), and a second derivative contained this mutation combined with three additional mutations, namely NS1[S5T], N[M50L] and L[N1249K] (Min AL-M2-1[E70D]+3).

All seven Min AL-derived viruses were readily rescued by reverse genetics and Sanger sequencing confirmed that the working virus stocks were free of any adventitious mutations.

### Mutations identified in P14 of the *in vitro* stress test increased multi-cycle Min AL replication *in vitro*

We evaluated the effects of the introduced P14 mutations on the multicycle replication of Min AL at 32°C and 37°C. Vero cells were infected with an MOI of 0.01 pfu/cell and virus replication was monitored for 10 to 12 days (Fig 3B and 3C). Due to the large number of viruses to evaluate, two separate multi-cycle replication experiments were done. Min AL-derived viruses containing mutations from lineage #1 or #7 as well as the corresponding P14 of lineage #1 and #7 were evaluated in the same experiment (Fig 3B and 3C, left and right panels), while Min AL-derived viruses containing mutations from lineage #3 as well as P14 of lineage #3 were evaluated in a separate experiment (Fig 3B and 3C, center panels). Wt RSV and Min AL were included in both experiments.

At 32°C (Fig 3B), wt RSV reached peak titers of 6.9–7.1 log_10_ pfu/ml on days 11–12 pi. Replication of Min AL was delayed and was maximal on day 12 pi (6.2–6.4 log_10_ pfu/ml). Min AL-M2-1[I87K] from lineage #1 and all three Min AL-derived viruses from lineage #3 replicated to higher titers than Min AL, achieving peak titers similar to or surpassing those of wt RSV (6.9 to 7.5 log_10_ pfu/ml). Min AL-M2-1[I87K]+2 and viruses from lineage #7 replicated to similar titers as Min AL (6.2 log_10_ pfu/ml).

At 37°C (Fig 3C), differences were much more pronounced. Wt RSV reached peak titers of 6.4 to 7.2 log_10_ pfu/ml. In contrast, Min AL replicated poorly, reaching titers below 2.5 log_10_ pfu/ml. The incremental reintroduction of the selected P14 mutations increased replication of Min AL incrementally in the case of lineages #3 and #7. The individual mutations M2-1[I87K], P[G26D], and M2-1[E70D] from lineage #1, #3 and #7, increased Min AL titers by 128-, 9-, and 54-fold, respectively. Reintroduction of the additional set of mutations from each lineage recovered Min AL replication incrementally to P14 levels in the case of lineages #3 and #7. This showed that these mutations indeed were responsible for the partial loss of the ts phenotype of these Min AL lineages.

To determine whether the introduced P14 mutations reduced the temperature sensitivity of Min AL, we evaluated their effect on the T_SH_ of Min AL in a plaque assay on Vero cells at temperatures ranging from 32°C to 40°C (Table 2). This showed that, indeed, the incremental reintroduction of the P14 mutations from lineages #1 and #7 increased the T_SH_ of Min AL from 36°C to 39°C, and the reintroduction of the P14 lineage #3 mutations increased the T_SH_ to 40°C, confirming the compensatory effect of the P14 mutations on the ts phenotype of Min AL.

**Table 2:** Temperature sensitivity of Min AL and derivatives on Vero cells.

		Virus titer (log_10_ PFU per ml) at indicated temperature (°C)^a^							
	**Virus**	**32**	**35**	**36**	**37**	**38**	**39**	**40**	**T**_**SH**_
	Min AL	7.1	5.6	**4.3**	3.2	1.9	<1	<1	**36**
Lineage #1	Min AL [I87K]	6.8	6.6	5.2	**4.6**	3.8	3.0	<1	**37**
	Min AL [I87K]+2	6.8	6.4	6.3	6.0	5.1	**3.4**	<1	**39**
Lineage #3	Min AL [G26D]	7.2	6.7	6.2	5.7	**4.1**	2.5	2.0	**38**
	Min AL [G26D]+2	7.1	7.1	6.6	6.2	**5.0**	3.4	2.2	**38**
	Min AL [G26D]+3	7.0	7.0	6.8	6.7	6.5	5.4	**2.6**	**40**
Lineage #7	Min AL [E70D]	6.7	5.8	**4.5**	4.2	3.8	2.3	<1	**36**
	Min AL [E70D]+3	6.2	6.1	5.5	5.4	5.2	**2.4**	1.4	**39**
	Wt RSV	7.6	7.5	7.5	7.4	7.5	7.4	7.3	**>40**

^a^ For viruses with a *ts* phenotype, the shut-off temperatures (T_SH_) are underlined and listed in boldface at the right. See S1 Table for definition of T_SH_. The *ts* phenotype is defined as having a T_SH_ of 40°C or less.

### Single cycle virus replication experiments to analyze the effects of P14 mutations on viral RNA, protein synthesis, and viral yield by Min AL

**Fig 4:**
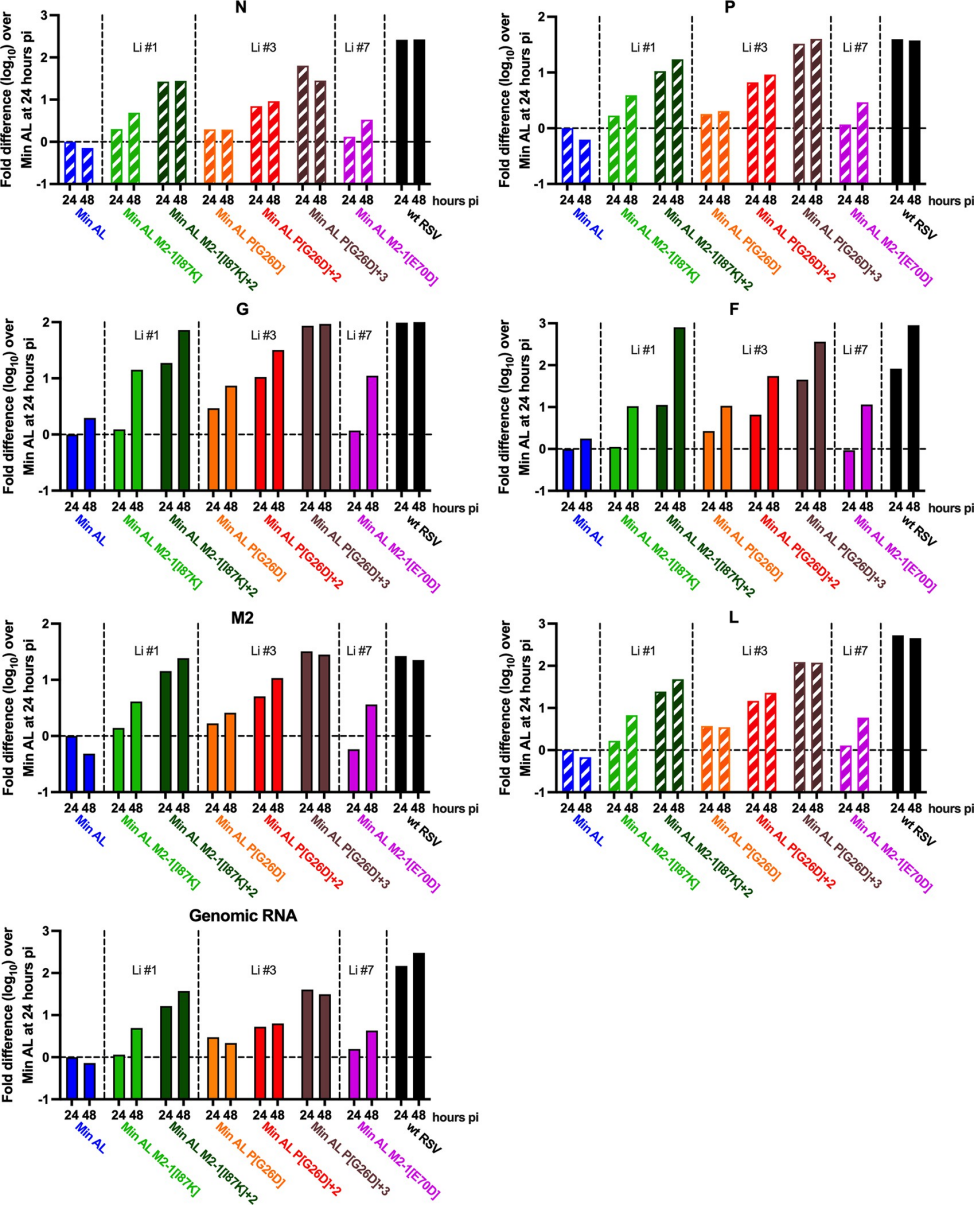
Prominent mutations identified from the *in vitro* stress test incrementally rescued RNA synthesis by Min AL. Vero cell monolayers in six-well plates were inoculated with an MOI of 3 pfu/cell with the indicated viruses at 37°C and the total cell-associated RNA was harvested at 24 and 48 hpi. Positive-sense RSV RNAs (primarily mRNA, with a small content of antigenome) were quantified in triplicate by strand-specific RT-qPCR using tagged primers (Materials and Methods). Data for the wt and CPD ORFs are shown with solid and hatched bars, respectively. Note that the sequences of the CPD ORFs (N, P, L) differed from the wt ORFs and therefore necessitated the use of separate primers and probes. Thus, taqman results involving the CPD N, P and L ORFs of the Min AL-derived viruses could not be directly compared to those of wt RSV. The accumulation of negative-sense genomic RNA also was evaluated using a strand-specific RT-qPCR assay with tagged primers and a probe specific to the M2-1 ORF, which was the unchanged wt sequence in all viruses and permitted direct comparisons. Data were normalized to 18S ribosomal (r)RNA and expressed as log_10_ fold increase over Min AL at the 24 hour pi time point.

**Fig 5:**
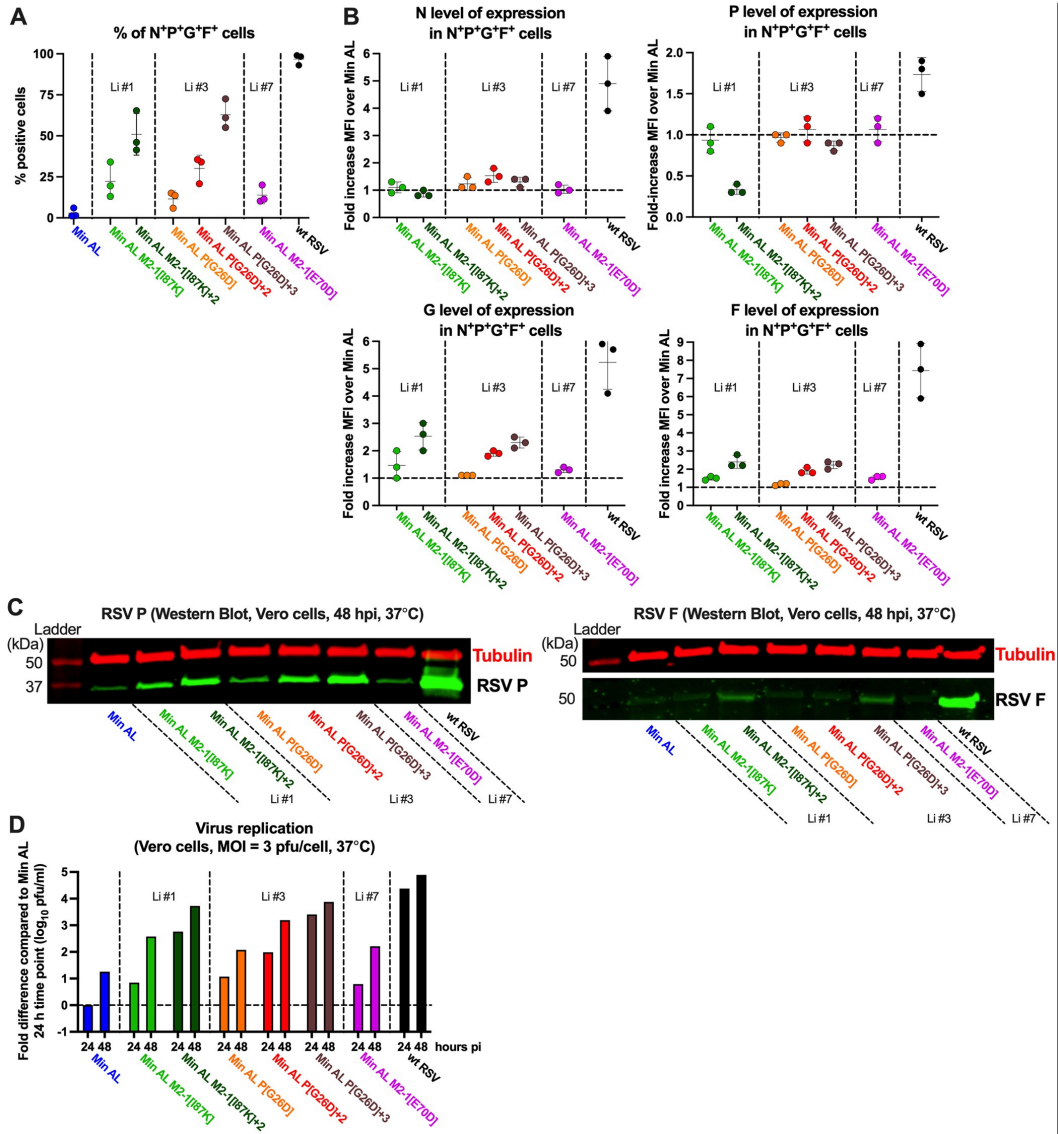
Prominent mutations identified from the *in vitro* stress test incrementally and partially rescued protein expression and virus production by Min AL. **A-C.** Protein expression. Additional replicate Vero cell monolayers from the single-cycle infection experiment described in Fig 4 (MOI of 3 pfu/cell, 37°C) were harvested at 48 hpi (one well per virus per time point) for analysis of viral protein expression by flow cytometry (A and B) and Western blot (C). (A and B) show data from the experiment in Fig 4 combined with two additional independent repeat experiments. Cells were permeabilized, immunostained for RSV N, P, G, and F protein expression, and evaluated by flow cytometry to determine the percentage of cells expressing the N, P, G and F proteins (A) as well as the level of expression of N, P, G and F proteins (expressed as median fluorescence intensity; MFI) in the N^+^P^+^G^+^F^+^ cells (B). Each experiment is represented by a symbol and means and standard deviations are shown. (C) Cell lysates were prepared and analyzed by Western blotting to evaluate the expression of P and F with tubulin used as a loading control. Ladder, molecular weight marker. **D.** Virus production. Additional replicate Vero cell monolayers from the experiment described in Fig 4 were harvested by scraping at 24 and 48 hpi (one well per virus per time point), vortexed, clarified, and titered in duplicate by immunoplaque assay to determine virus titer.

We next evaluated the effect of the P14 mutations on the expression of cell-associated viral RNA and proteins, as well as single-cycle viral yield, by Min AL derivatives during single-cycle replication experiments. These experiments were performed at 37°C (Figs 4 and 5), the temperature that had been shown in Fig 1C and 3C to have substantial differential effects on the replication of Min AL and its derivatives. To do so, quadruplicate wells of Vero cells per time point were infected with an MOI of 3 pfu/cell with wt RSV, Min AL, or the Min AL-derived viruses and incubated at 37°C. Due to the low titer of Min AL M2-1[E70D]+3 stock (representing lineage #7), this virus was not included in these evaluations. At 24 and 48 hpi, one well was harvested for total cell-associated RNA in order to evaluate the accumulation of positive- and negative-sense viral RNA (Fig 4); at 48 hpi, one well was used to harvest cells and evaluate viral protein expression by flow cytometry (Fig 5A and 5B); at 48 hpi, one well was used to collect cell lysates and evaluate protein expression by Western blot analysis (Fig 5C); and at 24 and 48 hpi, one well was used to harvest cell suspensions that were vortexed, clarified, and evaluated by plaque assay to measure single-cycle viral yield (Fig 5D). The results are described in the next two sections.

### Single cycle virus replication: P14 mutations increased the accumulation of positive-sense viral RNAs by Min AL to wt RSV levels

From the single-cycle replication experiment described above (using Vero cells infected with an MOI of 3 pfu/cell at 37°C), viral RNA was extracted from cell lysates at 24 and 48 hpi, and gene-specific RT-qPCR taqman assays were used to quantify the accumulation of positive-sense N, P, G, F, M2 and L RNA as well as negative-sense genomic RNA (Fig 4). Note that intracellular positive-sense RSV RNA at these time points generally contains approximately 95% mRNA and 5% antigenomic RNA, and thus this assay was primarily for viral transcription. Because the nt sequences of the CPD and wt ORFs of the N, P, and L gene differed due to the CPD, different taqman assays had to be used to quantify CPD RNAs (Fig 4, hatched bars) and wt RNAs (solid bars), thus preventing direct comparison between Min AL and its derivatives with wt RSV based on these three genes. However, direct comparison between all viruses was possible for the G, F and M2 genes because they were unchanged in all viruses, as well as for the genomic RNA, which was quantified based on the M2 sequence that also was unchanged in all viruses.

Cells infected with Min AL at 37°C had low levels of accumulation of the various positive-sense viral RNAs at 48 hpi, indicating that the Min AL polymerase complex was inefficient at this temperature. For the other viruses at 24 and 48 hpi, the level of accumulation of each RNA was normalized relative to the Min AL value at 24 hpi taken as 1.0 (Fig 4). This analysis showed that the incremental addition of P14 mutations from lineages #1, 3, or 7 to Min AL increased the expression of all of the evaluated CPD and wt RNAs, and the complete sets of major mutations from lineages #1 (Min AL-M2-1[I87K]+2) or #3 (Min AL-P[G26D]+3) had the strongest effects. Indeed, at 48 hpi, the levels of positive sense G, F and M2 RNA in cells infected with Min AL-M2-1[I87K]+2 (lineage #1) or Min AL-P[G26D]+3 (lineage #3) were between 24- and 800-fold increased compared to Min AL at 24 hpi, and were comparable to wt RSV. This indicated that the sets of major P14 mutations increased the accumulation of positive-sense mRNAs by Min AL derivatives to levels that approached or equaled those of wt RSV at 24 hpi and, especially, at 48 hpi. In case of lineage #7, we were only able to compare the effect of the M2-1[E70D] mutation to wt RSV (because of the low titer of the other Min AL derivative bearing lineage #7 mutations). Its addition to Min AL moderately increased the synthesis of the positive-sense RNAs, similarly to the increases observed after addition of M2-1[I87K], an M2-1 mutation identified in lineage #1.

We also evaluated the accumulation of cell-associated negative-sense genomic RNA, representative of RNA replication. Accumulation of genomic RNA was undetectable between 24 and 48 hpi in cells infected with Min AL. Similarly to the positive-sense RNA, the incremental addition of the selected P14 mutations from lineages #1, 3, and 7 into Min AL increased the accumulation of negative-sense genomic RNA, and the complete sets of major mutations from lineage #1 or #3 had the strongest effect (30-fold increase compared to Min AL). However, the levels of genomic RNA in cells infected with Min AL-derived viruses remained 8- to 10-fold lower than in wt RSV-infected cells.

### Single-cycle virus replication: P14 mutations increased Min AL protein expression and viral yield

From the single-cycle virus replication experiments described above (using Vero cells infected with an MOI of 3 pfu/cell at 37°C), we also evaluated the effects on Min AL of the P14 mutations on cell-associated viral protein expression and single-cycle viral yield. Viral protein expression was evaluated at 48 hpi by flow cytometry (Fig 5A and 5B). Data in Fig 5A and 5B are derived from additional wells in the experiment described in Fig 4 as well as from two additional independent experiments (n = 3 total).

For flow cytometry analysis, the infected Vero cells were harvested at 48 hpi, then fixed and stained under permeabilizing conditions with a cocktail of fluorochrome-labeled monoclonal antibodies to evaluate the expression of N, P, G and F proteins. Analysis was done on live single N^+^P^+^F^+^G^+^ cells.

At 48 hpi, only 3% on average of the cells infected with Min AL were positive for all four proteins by flow cytometry (Fig 5A). Similar to the viral gene expression, the incremental addition of P14 mutations from each lineage into Min AL increased the frequency of cells expressing all four viral proteins. The addition of the full set of selected P14 mutations from lineage #1 or #3 had the strongest effect, resulting in 51% to 63% of the cells expressing all four proteins. This remained lower than wt RSV, for which 97% of cells expressed all four viral proteins.

We also determined the median level of expression of N, P, G and F protein in N^+^P^+^G^+^F^+^ cells at this 48-hpi time point (expressed as median fluorescence intensity; MFI) (Fig 5B). Since the levels of viral mRNAs in cells infected with Min AL-derived viruses bearing the complete set of lineage #1 or #3 mutations had been found to approach or equal the levels for wt RSV at 48 hpi (Fig 4), it was of particular interest to determine whether the levels of expression of the viral proteins similarly approached or equaled those of wt RSV. In general, at 48 hpi, the MFIs of all four viral proteins remained substantially lower in cells infected with Min AL or derivatives than in cells infected with wt RSV (note that in Fig 5B, the level of expression of Min AL is set at 1.0 and values for the Min AL derivatives and wt RSV are expressed as fold-increases). For example, the MFI of the RSV N protein was about 5-fold lower in Min AL-infected cells than in wt RSV-infected cells, and the addition of lineage #1, 3, or 7 P14 mutations to Min AL did not substantially increase the N MFI closer to wt RSV levels; similarly, the MFI of the RSV P protein was about 2-fold lower in cells infected with Min AL or its derivatives compared to cells infected with wt RSV. Thus, while a higher proportion of cells was infected by Min AL derivatives compared to Min AL, the level of expression of N and P in individual cells was similar among Min AL viruses, and lower than in wt RSV infected cells. This might be due to the inefficiency of protein translation from CPD mRNAs.

Interestingly, in cells infected with the Min AL M2-1[I87K]+2 virus, which included the c2334a mutation in the P gene start signal, P protein expression was further reduced to less than half of that of Min AL (Fig 5B, see Discussion). In the case of the G and F proteins, which were encoded by ORFs not modified by CPD, the incremental addition of mutations from each lineage into Min AL increased the protein expression in infected cells by about 2- to 2.5-fold for the viruses that contained all the mutations from lineage #1 or #3. However, G and F expression still remained lower compared to wt RSV infected cells that showed 5- to 7-fold higher G and F expression than Min AL-infected cells.

From the single-cycle replication experiment described above, additional wells were harvested at 48 hpi for Western blot analysis using monoclonal antibodies against the RSV F, P (Fig 5C) and G (S1 Fig). G was only detected in Min AL P[G26D]+3 and wt RSV-infected cells and was predominantly detected as a diffuse band between 100 and 150 kDa, corresponding to the full-length, fully-glycosylated form. A few less-abundant bands (30–70 kDa) were also detected in wt RSV infected cells. These might represent intermediates with incomplete O-glycosylation or truncated versions of the G protein that are mostly present on virions released from infected Vero cells [38]. The blots were quantified by scanning, and the observed levels of P, F and G proteins were consistent with the flow cytometry results of Fig 5A and 5B.

Single-cycle viral yields of Min AL and derivatives were also evaluated. The incremental addition of mutations from each lineage to Min AL increased virus replication, following a trend comparable to that of the accumulation of viral genomic RNA and protein expression. However, the viral titers of the Min AL derivatives were at least 10-fold less at both time points compared to wt RSV.

### Min AL and derivatives are highly attenuated in hamsters

**Fig 6:**
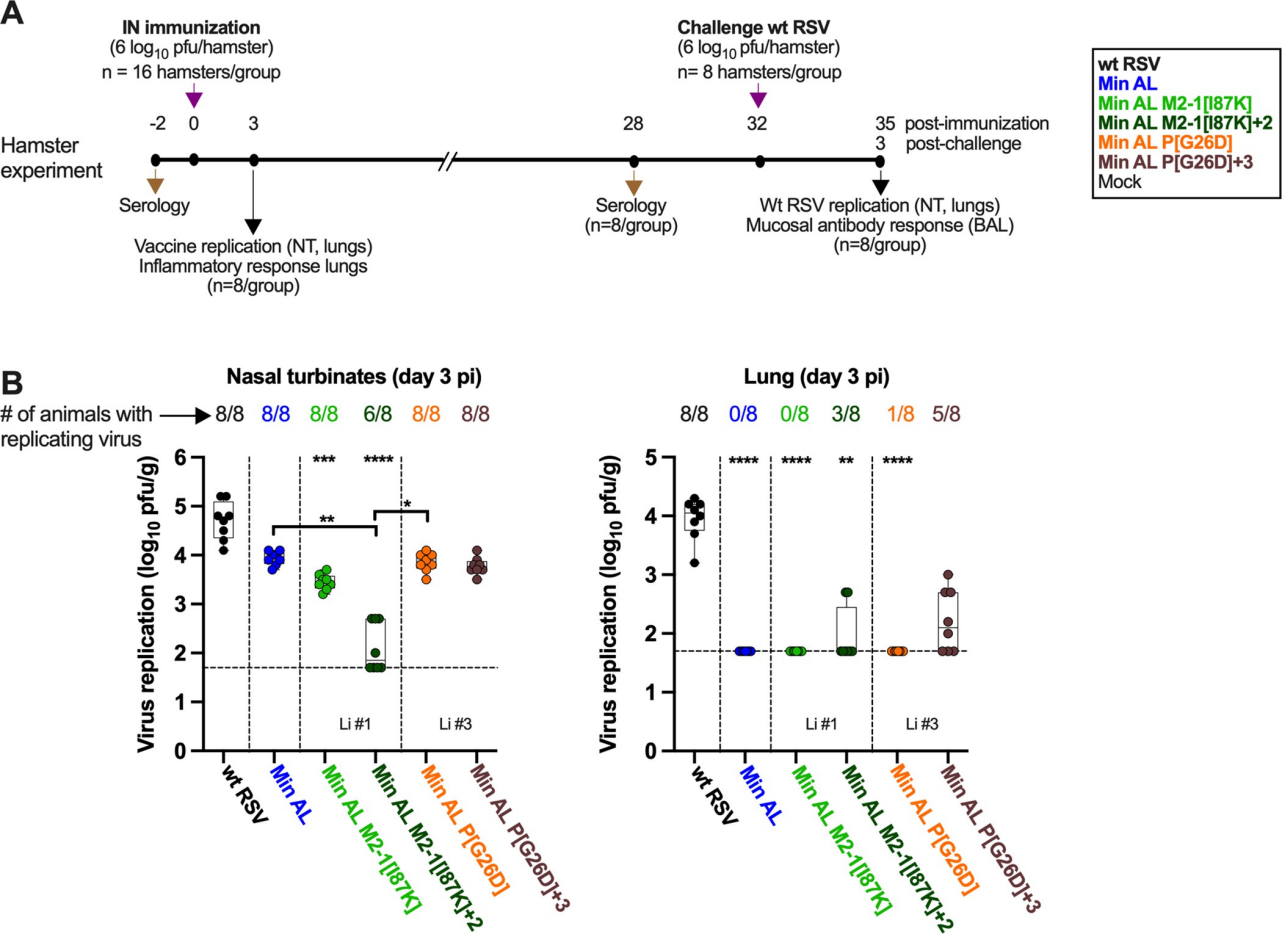
Replication of Min AL and derivatives following IN inoculation of hamsters. **A.** Timeline of the hamster experiment. Groups of 16 five- to six-week old golden Syrian hamsters were inoculated IN with 6 log_10_ pfu per hamster of the indicated virus. An additional group of eight animals were kept uninfected as controls. At 3 days post-infection (dpi), eight hamsters per group were euthanized and nasal turbinates (NT) and lungs were harvested and homogenized, and viral titers were determined by immunoplaque assay. Serum was collected at day -2 and 28 dpi for evaluation of the anti-RSV antibody response. At 32 dpi, all remaining hamsters including the group of eight non-immunized control hamsters were challenged with wt RSV. At 3 days post challenge (dpc), all hamsters were euthanized, bronchoalveolar lavages (BAL) were collected for evaluation of the mucosal antibody response in the lower airways and NT and lungs were harvested for evaluation of the replication of the challenge wt RSV virus. **B.** Replication of wt RSV, Min AL and derivatives in NT and lungs at 3 dpi from eight hamsters per group. Harvested tissues were homogenized, clarified, aliquoted, snap frozen in dry ice and stored at -80°C. Titers were determined by immunoplaque assay and expressed as pfu/g of tissue. The limit of detection is 50 pfu/g of tissue (dotted line). Viruses are grouped by lineage (Li), i.e., the number of the *in vitro* stress test lineage in which the specific mutations were identified. The median, min, and max values, 25^th^ and 75^th^ quartile, and individual values are shown. Statistical differences in comparison to wt RSV are indicated at the top of each graph, while differences between Min AL and its derivatives are indicated in brackets (*p ≤ 0.05; **p ≤ 0.01; ***p ≤ 0.001; ****p ≤ 0.0001, Kruskall Wallis test with Dunns post hoc test).

We next evaluated the replication, immunogenicity, and protective efficacy of Min AL and derivatives in hamsters (see Fig 6A for the timeline of the experiment). Due to low titers of virus stocks, three Min AL derivatives were not included for analysis: Min AL P[G26D]+2 (lineage #3), Min AL M2-1[E70D] (lineage #7), and Min AL M2-1[E70D]+3 (lineage #7). Groups of 16 golden Syrian hamsters were inoculated IN with 6.0 log_10_ pfu of the indicated virus. Hamsters did not show any clinical signs or symptoms of respiratory illness after inoculation with wt RSV, Min AL or Min AL derivatives, as typical for RSV in the hamster model [39]. Virus replication in nasal turbinates (NT) and lungs was evaluated from eight hamsters per group on day 3 pi. Lungs appeared, as expected, normal without gross pathology.

In the NT, wt RSV replicated efficiently to a geometric mean titer (GMT) of 4.7 log_10_ pfu/g (Fig 6B, left panel). Replication of Min AL was reduced by about 10-fold compared to wt RSV. Interestingly, the restriction of replication of Min AL increased with the incremental addition of the lineage #1 mutations. Specifically, introduction of mutation M2-1[I87K] alone (virus Min AL-M2[I87K]) reduced Min AL titers by about 3-fold and the further addition of the NS1[I46M] and P gene start (c2334a) mutations (virus Min AL-M2[I87K]+2) reduced Min AL replication by about 70-fold (p<0.01 compared to Min AL). The introduction of the lineage #3 mutations (viruses Min AL-P[G26D] and Min AL-P[G26D]+3) did not affect replication of Min AL in NT.

In the lungs, wt RSV replicated to a GMT of 3.9 log_10_ pfu/g, as typically observed (Fig 6B, right panel). No or baseline replication of Min AL, Min AL M2-1[I87K] (lineage #1) and Min AL P[G26D] (lineage #3) was detected, showing that Min AL and derivatives were highly attenuated in the lung (p<0.0001 compared to wt RSV). However, the inclusion of further mutations from lineage #1 (Min AL M2-1[I87K]+2) and #3 (Min AL P[G26D]+3) partly rescued Min AL replication, with virus replication detectable in the lungs of 3 of 8 hamsters and 5 of 8 hamsters, respectively.

We also evaluated the expression of 13 inflammation-related genes by RT-qPCR at day 3 pi in the lungs of seven of the eight hamsters that were inoculated with Min AL or wt RSV (S2 Fig). Data are expressed as fold-increase over the expression determined from two unimmunized hamsters. This showed that, although wt RSV replicated to approximately 3.9 log_10_ pfu/g in the lungs on day 3 (Fig 5B), it induced only a moderate inflammatory response, as only the interferon-induced gene MX-2 and the pro-inflammatory cytokine IL-1β were increased in lung tissues (10- and 5-fold increase compared to unimmunized animals, respectively). Min AL did not replicate to detectable levels in the lungs, as shown in Fig 5B, and the level of expression of these two cytokines was lower in Min AL-inoculated animals (p<0.05 compared to wt RSV for the expression of MX-2; S2 Fig).

### Min AL and derivatives induce robust serum anti-RSV antibodies

**Fig 7:**
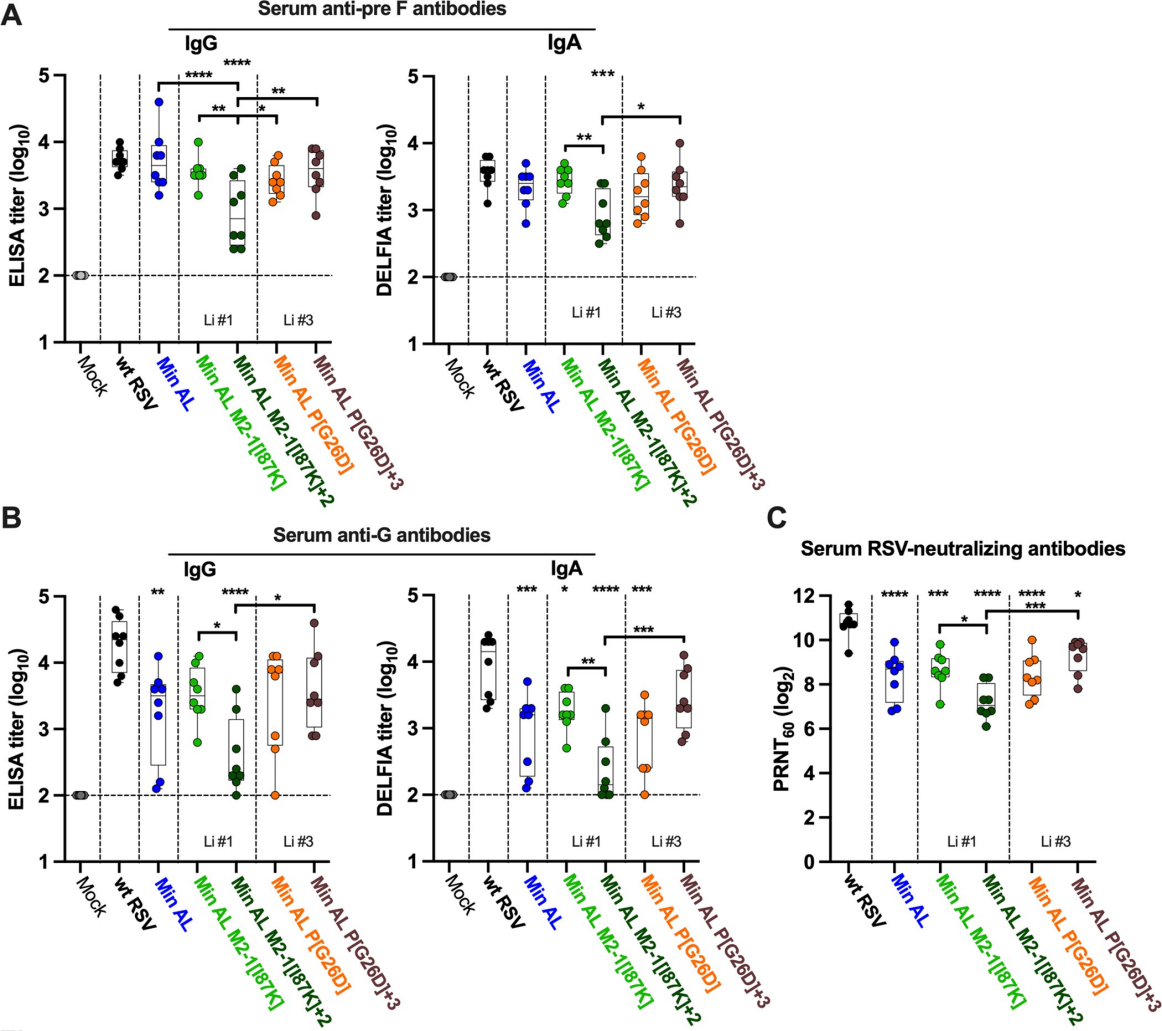
Min AL and derivatives induced robust serum anti-RSV antibody responses in hamsters. Sera from eight remaining hamsters per group including the group of non-immunized hamsters were collected at 28 dpi to evaluate the anti-RSV antibody response. **A-B.** Serum anti-RSV pre F (A) and G (B) IgG (left panels) and IgA (right panels) titers were determined by ELISA (IgG) or dissociation-enhanced lanthanide fluorescence immunoassay (DELFIA) ELISA (IgA). The limit of detection is two log_10_. **C.** The neutralizing activity of the sera from the immunized hamsters was evaluated by determining the 60% plaque-reduction neutralizing antibody titers (PRNT_60_), performed in the presence of complement. Viruses are grouped by lineage (Li), i.e., the number of the *in vitro* stress test lineage in which the specific mutations were identified. The median, min, and max values, 25^th^ and 75^th^ quartile, and individual values are shown. Statistical differences to wt RSV are indicated at the top of each graph, while differences between Min AL and its derivatives are indicated in brackets (*p ≤ 0.05; **p ≤ 0.01; ***p ≤ 0.001; ****p ≤ 0.0001, One-way ANOVA with Tukey post test).

**Fig 8:**
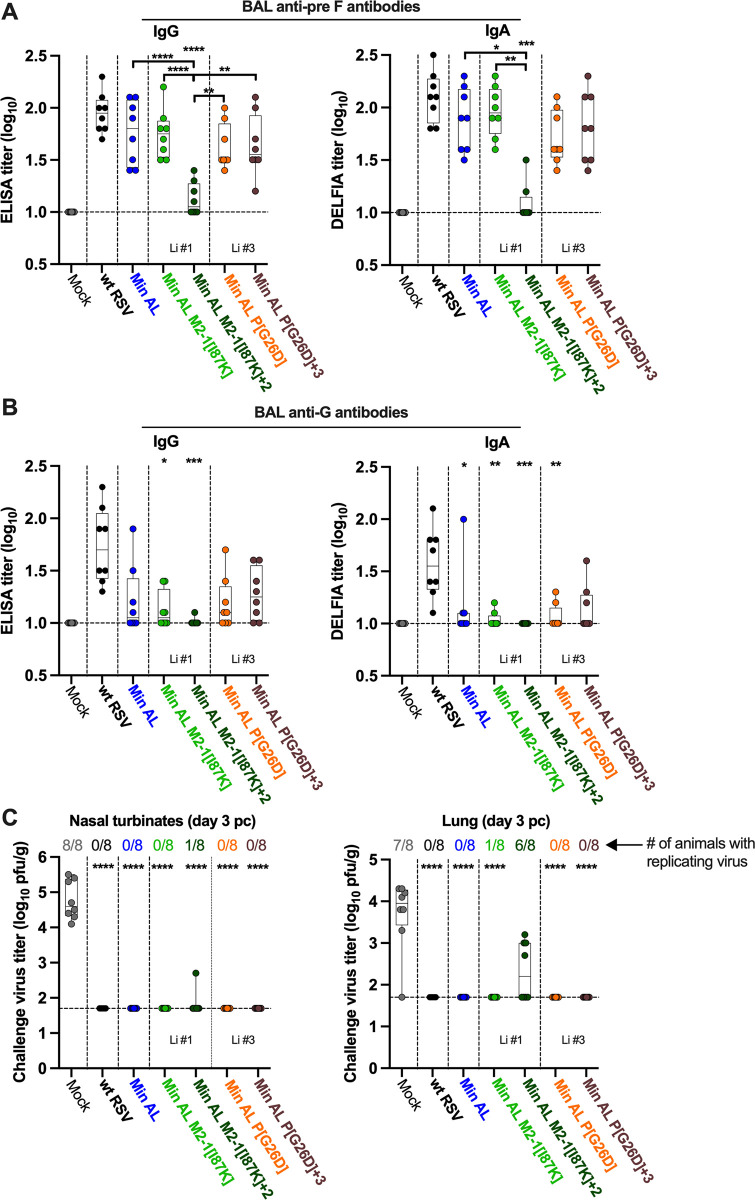
Min AL and derivatives induced robust mucosal antibody responses in the airways of hamsters and were protective against wt RSV challenge. At 32 dpi, eight hamsters per group including the group of eight non-immunized control hamsters were challenged IN with 6 log_10_ pfu of wt RSV. At day 3 pc, animals were euthanized and bronchoalveolar lavage (BAL), NT and lung tissues were collected from each animal. **A-B.** Anti-RSV pre F (A) and G (B) IgG (left panels) and IgA (right panels) titers in BAL were determined by ELISA (IgG) or DELFIA ELISA (IgA). The limit of detection is one log_10_. **C.** Replication of wt RSV challenge virus was evaluated by plaque assay from NT or lung tissues harvested at day 3 pc. In each graph, the median, min, and max values, 25^th^ and 75^th^ quartile, and individual values are shown. Viruses are grouped by lineage (Li), i.e., the number of the *in vitro* stress test lineage in which the specific mutations were identified. Statistical differences to wt RSV are indicated at the top of each graph, while differences between Min AL and derivatives are indicated in brackets (*p ≤ 0.05; **p ≤ 0.01; ***p ≤ 0.001; ****p ≤ 0.0001, Kruskall Wallis test with Dunns post hoc test or One-way ANOVA with Tukey post test).

We next evaluated the immunogenicity of Min AL and derivatives (Figs 7 and 8). In the hamster experiment described above, sera were collected at day 27 pi from the eight remaining hamsters per group and the levels of IgG and IgA antibodies binding to the prefusion form of RSV F (pre F) or RSV G were determined by ELISA using recombinantly-expressed purified pre F and G proteins (Fig 7).

Surprisingly, despite the strong restriction of replication in hamsters, Min AL induced high titers of serum anti-pre F IgG and IgA that were comparable to those induced by wt RSV (Fig 7A). With the exception of the most attenuated Min AL-derived virus (Min AL M2-1[I87K]+2), which induced the lowest titers of anti-pre F IgG and IgA antibodies, the other Min AL derivatives induced high titers of serum anti-pre F IgG and IgA that also were comparable to those induced by wt RSV. Min AL and derivatives also induced robust levels of serum anti-RSV G IgG and IgA antibodies, however the titers induced by Min AL were significantly lower than those induced by wt RSV (p<0.01 and p<0.001 for IgG and IgA, respectively, Fig 7B). Among the Min AL derivatives, Min AL M2-1[I87K] and Min AL P[G26D]+3 induced higher titers of serum anti-RSV G IgG and IgA than Min AL, whereas the titers induced by Min AL P[G26D]+3 were not significantly different than those induced by wt RSV.

We also determined the serum 60%-plaque-reduction RSV-neutralizing antibody titers (PRNT_60_) induced by the viruses (Fig 7C). Min AL and derivatives induced robust mean PRNT_60_ ranging from 7.2 to 9.3 log_2_ (for Min AL M2-1[I87K]+2 and Min AL P[G26D]+3, respectively) albeit these titers were significantly lower than those induced by wt RSV (10.8 log_2_; p<0.05 to p<0.0001).

### Min AL and derivatives induced robust mucosal anti-RSV antibodies and were fully protective against wt RSV challenge

In the hamster experiment described above, the remaining hamsters were challenged on day 32 with 6.0 log_10_ pfu of wt RSV (Fig 6A). Similarly to our observations after immunization, hamsters did not show any clinical signs or symptoms of respiratory illness after challenge with wt RSV. At day 3 post challenge (pc), all animals were necropsied and bronchoalveolar lavages (BAL) were collected to evaluate the levels of anti-RSV pre F and G IgG and IgA binding antibodies in the lower airways (Fig 8A and 8B). As expected, lungs appeared normal without gross pathology. NT and lung tissues were collected, homogenized, and subjected to immunoplaque assay to determine the level of replication of challenge wt RSV (Fig 8C).

Remarkably, although the replication of Min AL and derivatives had been low or undetectable in the lungs of immunized hamsters, as described above, these viruses (with the exception of the most attenuated Min AL M2-1[I87K]+2 virus) induced robust titers of anti-RSV pre F IgG and IgA in the lungs that were not significantly different than to those induced by wt RSV (Fig 8A). Similarly to the serum, Min AL and derivatives induced detectable but lower levels of anti-G IgG and IgA than wt RSV (Fig 8B). As expected, no anti-RSV pre F and G IgG and IgA antibodies were detected in the lungs of control mock-immunized hamsters that were challenged with wt RSV (Fig 8A and 8B).

We next evaluated the protective efficacy of Min AL and derivatives against wt RSV challenge at day 3 pc (Fig 8C). Wt RSV replicated efficiently in the NT and lung tissues of hamsters from the mock-immunized group. However, with the exception of Min AL M2-1[I87K]+2, the most-attenuated virus, Min AL and the other derivatives were fully protective against detectable wt RSV challenge replication in NT and lung tissues. To confirm that Min AL-immunized hamsters were protected against inflammatory responses after challenge, we also evaluated the expression of 13 inflammation-related genes by RT-qPCR on day 3 pc. We selected lungs from 7 mock-immunized animals with robust RSV challenge virus replication, and 7 Min AL-immunized animals (no detectable challenge virus replication in this group). Data are expressed as fold-increase over the expression determined from two unimmunized unchallenged hamsters [40]. Similarly to the results from S2 Fig, only the interferon-inducible gene Mx-2 and the pro-inflammatory cytokine gene IL-1β were increased in mock-immunized animals on day 3 after RSV challenge (9- and 3-fold increase compared to unimmunized unchallenged animals, respectively; p<0.001 and p<0.01 for Mx-2 and IL-1β; S3 Fig), indicative of only a moderate inflammatory response in mock-immunized animals after RSV challenge. However, in Min AL-immunized animals, the level of expression of the 13 inflammation-related genes after wt RSV challenge was comparable to unimmunized unchallenged animals, indicating that Min AL was protective against RSV challenge, with no sign of inflammation after challenge.

### Min AL-derived viruses are genetically and phenotypically stable *in vitro*

**Fig 9:**
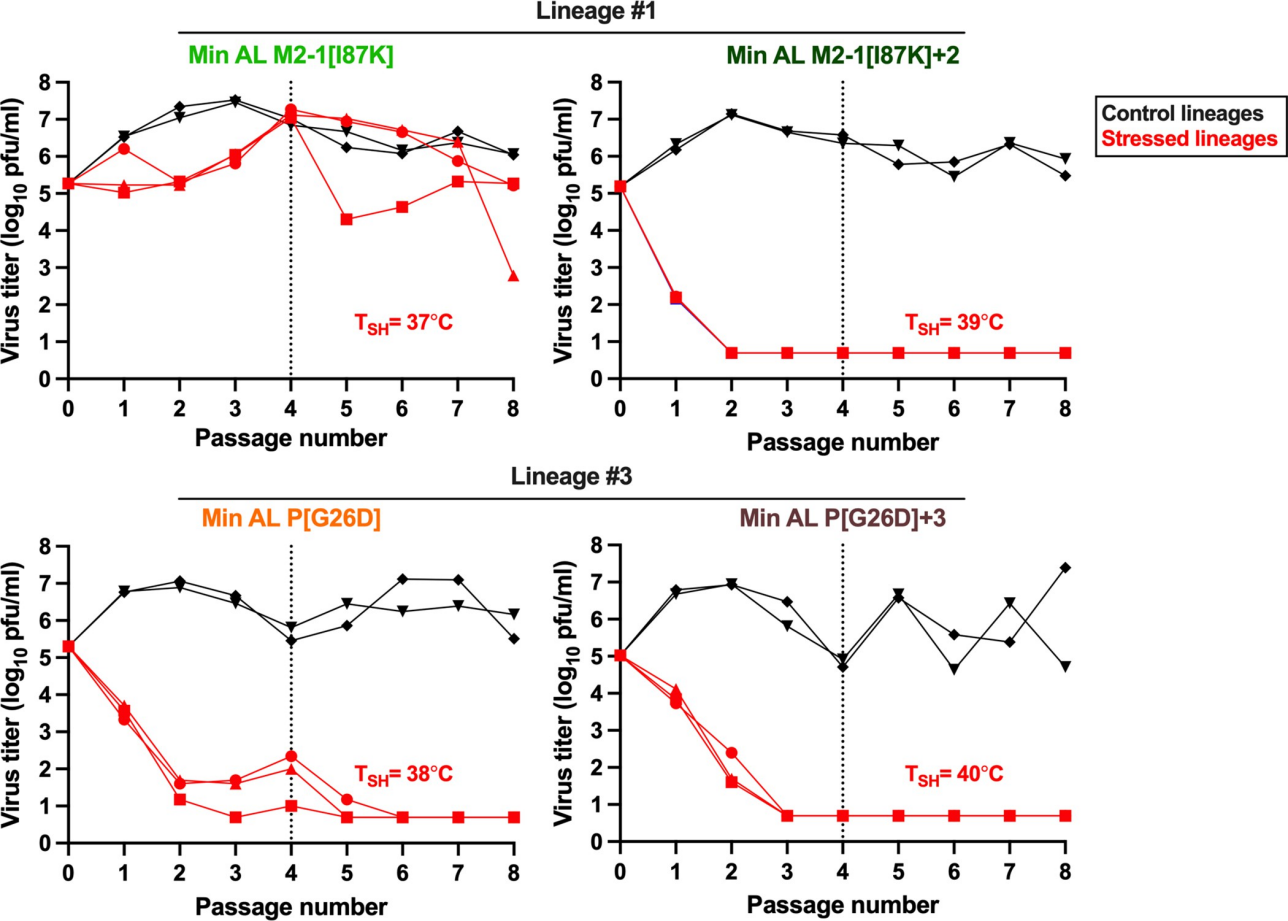
Min AL derivatives were genetically stable during an *in vitro* stress test. The stability of four Min AL-derived viruses (Min AL M2-1[I87K], Min AL M2-1[I87K]+2, Min AL P[G26D] and Min AL P[G26D]+3) was evaluated in an *in vitro* stress test. Five replicate cultures of Vero cells in 25 cm^2^ flasks were inoculated using an initial MOI of 0.1 pfu/cell. Two replicate cultures (in black) were passaged eight times at the permissive temperature of 32°C. Three replicate cultures (in red) were incubated for four passages at one degree below the indicated virus T_SH_, followed by four additional passages at the virus T_SH_, representing two months of culture. Each lineage is represented by a different symbol. Flasks were harvested when extensive syncytia were observed or when the cells started to detach. One of five ml of clarified fluids from the previous passage was used to infect the following passage of fresh cells. Furthermore, aliquots of clarified virus from each lineage were snap frozen for virus titration by plaque assay at 32°C at the end of the experiment and sequencing. Whole genome Sanger sequencing was performed at the end of P8 for the lineages that were passaged at 32°C. Whole genome Sanger sequencing of two of three Min AL M2-1[I87K] lineages (upper left panel, red square and circle) was done at the end of P8 while sequencing of the third lineage (red triangle) was done at P7 instead of P8 due to low virus titer at P8. Due to low replication, whole genome sequencing of the other three stressed Min AL derived viruses could not be done, but the sharp drop in replication was indicative of stability.

Finally, we evaluated the genetic stability and the stability of the temperature-sensitive phenotype of the four Min AL-derivatives that were evaluated in the hamster experiment described above by subjecting them to an *in vitro* stress test (Fig 9). Five 25-cm^2^ flasks of Vero cells per virus were inoculated using an initial MOI of 0.1 pfu/cell. Three lineages per virus (in red) were passaged four times in parallel at a temperature that was one degree below the virus T_SH_ (indicated in Fig 9). The incubation temperature was increased by one°C (to the virus T_SH_) and four additional passages were made, for a total of eight passages, corresponding to approximately two months of continuous culture. The other two control lineages (in black) were passaged 8 times in parallel at the permissive temperature of 32°C.

All four Min AL-derived viruses replicated efficiently at the 32°C control temperature, reaching final titers at the end of P8 of between 4.7 and 7.4 log_10_ pfu/ml (Fig 9). Whole-genome Sanger sequencing using viral RNA isolated from supernatants harvested at P8 from all lineages at 32°C showed that none of the Min AL derivatives accumulated prominent mutations.

For the lineages passaged at increasing temperature (Fig 9), the Min AL M2-1[I87K] lineages replicated efficiently at one degree below its T_SH_ (37°C), reaching 7.0–7.3 log_10_ pfu/ml at P4 (upper left panel). The titers subsequently decreased during the four passages at the virus T_SH_. Whole-genome Sanger sequencing of viral RNA isolated from supernatants harvested at P7 for one lineage and at P8 for the two other lineages did not reveal any prominent mutations. The three other Min AL-derivatives, (Min AL-M2-1[I87K]+2, Fig 9, upper right panel; Min AL P[G26D], lower left panel; and Min AL P[G26D]+3, lower right panel), exhibited strongly reduced replication when passaged at one degree below their respective T_SH_ and were completely restricted at their respective T_SH_, indicating that they did not acquire mutations that rescued replication. Overall this experiment indicated that Min AL derivatives were genetically and phenotypically stable when serially passaged at the permissive temperature of 32°C and at their respective T_SH._

## Discussion

In the present study, we generated and evaluated a new CPD RSV vaccine candidate, Min AL, which contained the CPD NS1, NS2, N, P, M, SH, and L ORFs of Min A combined with the CPD L ORF of Min L, while the G, F and M2 ORFs were kept unchanged. Min AL replicated efficiently at the permissive temperature of 32°C in Vero cells and was genetically stable when serially passaged at this temperature, which is an important consideration for vaccine manufacture. Similar to previous RSV derivatives containing CPD ORFs [33], Min AL was temperature sensitive: the T_SH_ of Min AL was 36°C, which is lower than that of Min A (40° C) or Min L (37°C), suggesting that Min AL had contributions to temperature-sensitivity from both parents. When serially passaged at increasing temperatures in an *in vitro* stress test, Min AL retained greater temperature-sensitivity compared to Min A and Min L: none of the Min AL lineages had any detectable replication at 40°C, a temperature at which the replication of stressed Min A lineages was barely affected and the replication of most of the Min L stressed lineages was reduced but still substantial for at least one passage [35,36].

Whole-genome deep sequencing of lineages of Min AL from P14 of the *in vitro* stress test revealed that, under temperature stress, Min AL acquired a diverse array of prominent missense point mutations occurring in four CPD ORFs (NS1, N, P, L) and four wt ORFs (G, F, M2-1, and M2-2). In previous studies, sequence analysis of passage levels of stress tests of Min L and Min A similarly identified many different mutations in both CPD and wt ORFs [35,36].

Despite the apparent diversity, the P14 mutations of the present study mostly occurred in genes encoding proteins of the polymerase/nucleocapsid complex. Four other missense mutations were in NS1, which previously was suggested by minigenome experiments to strongly affect RNA synthesis [41]. The greatest numbers of missense mutations were in P and M2-1. All P mutations were present in an N-terminal segment comprising aa 19–31 inclusive. Interestingly, missense mutations in this region of P were previously identified in stressed lineages of Min A and were found to rescue its replication at 40°C [35]. Each of the eight stressed Min AL lineages sequenced at P14 in the present study also contained one or more of M2-1 mutations. All M2-1 mutations were present in the N-terminal half of the protein, between aa 14 and 98, inclusive. These included mutations that were identical (A73S) or very similar (N88T, N88S) to mutations in M2-1 ([A73S] and [N88K]) that were identified previously during the stress test of Min L and were shown to rescue Min L replication at temperatures up to 40°C [36].

In previous molecular-dynamic simulations, mutations in the N-terminal domain of P were predicted to increase its flexibility, which might facilitate the interactions of P with N, resulting in increased efficiency of the RSV polymerase complex [35]. Also, previous molecular dynamic simulations of the [A73S] and [N88K] mutations identified in the M2-1 protein of stressed Min L lineages suggested that they increased the stability within or between M2-1 monomers, which also might increase the efficiency of the RSV polymerase complex [36]. We hypothesized that P14 mutations in polymerase components increase the activity of the polymerase on a per-molecule basis, and were selected during the *in vitro* stress test because they partly compensate for reduced viral gene expression due to CPD.

Interestingly, two prominent mutations were found in transcription gene-start signals. One was in the P gene-start signal (5^th^ nucleotide of the signal GGGGCAAAT). In a previous study, saturation mutagenesis of a gene-start signal in a minigenome showed that this mutation reduced transcription to 57% [42]. The second mutation was in the L gene-start signal (4^th^ nucleotide of the signal GGGACAAAAT) and the previous minigenome experiment indicated that A or G should be essentially equivalent at this position [42]. The sole mutation identified in one of the control lineages also involved a mutation in the P gene-start signal (8^th^ nucleotide of the signal GGGGCAAAT). In the previous minigenome experiment this mutation reduced mRNA synthesis by about 90% [42]. Thus, for lineages with a point mutation in non-ORF sequence, the mutation was in a transcription gene-start signal, and was in the P or L gene that encodes polymerase components. Since these mutations occur in transcription signals, and indeed in signals controlling transcription of two key components of the polymerase/nucleocapsid complex, they might serve to alter expression of these components in order to compensate for CPD. The reduced activity predicted for the two mutations in the P gene-start signal would be contrary to the idea that they cause increased expression of polymerase components. However, since the polymerase components in the Min AL derivatives also acquired missense mutations, the effects of the non-ORF mutations in the gene-start signals also may be different than the wt situation.

We performed a functional analysis of some the prominent missense mutations, as well as two gene-start mutations by introducing them in various combinations into the Min AL backbone. The reintroduction of single M2-1 or P mutations into Min AL resulted in a partial rescue of replication at 37°C. When additional mutations from the respective lineages were added, virus replication at 37°C was increased to the same level as the respective P14 pools from the *in vitro* stress test. This showed that these sets of mutations indeed were responsible for rescuing Min AL replication at increased temperatures, and that the largest sets of reintroduced mutations from lineage #3 and #7 gave full rescue to P14 levels.

The reintroduction of single M2-1 or P mutations into Min AL resulted in a partial rescue of expression of positive-sense RNA at 37°C. The addition of further P14 mutations increased the accumulation of positive-sense RNAs to levels approaching or equalling that of wt RSV. This supports the idea that these mutations increased the activity of the viral polymerase for the expression of positive-sense RNA, which mainly is mRNA. Since the M2-1 protein is generally known to function in transcription and not RNA replication, mutations in M2-1 in particular seem likely candidates to be involved in increasing the expression of positive-sense RNA.

In response to the incremental addition of P14 mutations to Min AL, the accumulation of cell-associated viral proteins was increased but remained substantially below that of wt RSV. Inefficient translation of CPD ORFs is one likely factor. Instability of CPD mRNAs is another potential factor, although the stabilities of CPD and non-CPD versions of viral genes could not be directly compared because the sequence differences necessitated different primers and probes. In addition, Min AL M2-1[I87K]+2 had a substantial reduction in accumulation of the P protein. This virus contained the c2334a mutation in the P gene-start signal, which reduced mRNA synthesis by 50% in a minigenome study [42], and thus might be responsible for the observed reduction in P protein expression. Although a corresponding reduction in positive-sense P RNA was not observed by RT-QPCR, this might reflect inefficient detection of P mRNA against the background of readthrough mRNAs and antigenome, or might indicate that the mutation acted at the level of translation. Another factor in the lesser accumulation of viral proteins is that the increase in viral mRNAs to levels comparable to wt RSV was more evident at 48 hpi than 24 hpi, and increases in the accumulation of viral proteins would be expected to lag temporally behind that of mRNAs and thus could be proportionately lower at 48 hpi. It is also possible that, due to their increased content of CpG and UpA, Min AL and its derivatives induced increased innate immune reactions *in vitro* that caused a global reduction in the synthesis of viral proteins. While increased innate responses were not observed for Min AL *in vivo* in the hamster experiment, the situation could be different for the Min AL derivatives *in vitro*.

The accumulation of negative-sense genomic RNA was increased by addition of the P14 mutations, with the full sets of mutations being more effective than the single M2-1 and P mutations. However, the level of accumulation of genomic RNA remained lower than that of wt RSV. This is not surprising, since RNA replication by non-segmented negative-strand RNA viruses depends on concurrent viral protein synthesis, which was reduced as discussed above. The production of infectious virus during single-cycle replication similarly was increased by the single multiple P14 mutations, and even more by multiple P14 mutations, but remained below the levels of wt RSV. This likely reflects the reduced accumulation of viral proteins and genomic RNA by the Min AL derivatives compared to wt RSV.

When inoculated IN into hamsters, Min AL replicated in the NT to levels comparable to those previously observed with Min A and Min L [35,36]. Surprisingly, the addition of the P14 mutations to Min AL, which had partially rescued replication of Min AL *in vitro* at 37°C, did not have any effect on replication in the NT or caused a decrease. In the lungs, Min AL did not replicate to detectable levels, while in previous studies Min A and Min L replicated at low levels [35,36]. The more attenuated phenotype of Min AL compared to Min A and Min L in the lungs likely reflects, at least in part, the increased temperature sensitivity of Min AL compared to its two parents (the body temperature of the Syrian hamster is 37°C). Increased temperature sensitivity is thought to preferentially restrict viral replication in the lower respiratory tract due to its higher temperature compared to the upper respiratory tract, and thereby is thought to provide additional safety in the lungs of infants. The greater attenuation of Min AL due to its increased temperature sensitivity compared to Min A and Min L is also supported by the increase in replication in the lungs by the addition of mutations that increase the T_SH_, such as in Min AL M2-1[I87K]+2 and Min AL P[G26D]+3, for which the T_SH_ was increased by 3°C and 4°C to 39°C and 40°C, respectively, compared to Min AL. Our *in vitro* stress tests involved virus replication under temperature stress over about 4 months of culture. However, in RSV-seronegative children and infants, replication of RSV in the respiratory tract is self-limiting, with virus clearance typically about 10 days after infection. While we cannot exclude that Min AL and derivatives might acquire missense mutations during this relatively brief period of replication in the airways of infants, the high extent of codon-pair deoptimization of the recoded virus and the further addition of stabilizing mutations into the genome of Min AL make it unlikely that the stabilized CPD RSV vaccine candidates will acquire mutations that will significantly increase their fitness during this short period of time.

Despite their high level of attenuation, Min AL and derivatives induced robust systemic and mucosal anti-pre F and anti-G IgG and IgA antibodies in hamsters. Surprisingly, with the exception of Min AL M2-1[I87K]+2, the levels of anti-pre F antibodies in both the serum and BAL of hamsters infected with Min AL or its derivatives were comparable to those induced by wt RSV. However, the levels of anti-G antibodies in both the serum and BAL were lower than those induced by wt RSV. The reason behind the differences between the levels of anti-pre F and anti-G antibodies is unknown and remains to be determined. The levels of serum anti-pre F and anti-G antibodies correlated well with the robust anti-RSV serum neutralizing antibody titers induced by Min AL and derivatives. Despite their greatly reduced levels of replication in hamsters, especially in the lungs, the systemic and mucosal antibody titers induced by Min AL derivatives were just slightly lower than those induced by wt RSV, suggesting that in some cases, the immunogenicity of the Min AL variants was stronger than expected based on their level of replication. One could speculate that the increase in CpG and UpA resulting from CPD may be immunostimulatory, compensating for some loss of immunogenicity due to the low level of replication of Min AL variants. We attempted to directly show this by evaluating the cytokine response in the lungs of hamsters inoculated with these candidates, but we were not able to detect cytokine induction in lungs of Min AL infected hamsters, likely due to the complete restriction of Min AL replication in the lower airways. Furthermore, with the exception of Min AL M2-1[I87K]+2 which was over-attenuated, Min AL and its derivatives were fully protective against challenge virus replication in both the upper and lower airways and no inflammation of the lungs of the immunized hamsters was detected after challenge. Lung pathology after challenge will also be evaluated in future studies.

In previous studies, the CPD RSVs that were attenuated *in vitro* were also attenuated in mice, hamsters and non-human primates. While there is no animal model available with a permissiveness for RSV similar to that of susceptible human hosts, RSV replicates to robust titers in the respiratory tract of hamsters following intranasal inoculation. Evaluation of these RSV vaccine candidates in hamsters generates useful preclinical information on their restriction in replication, providing a correlate for vaccine attenuation in the pediatric human target population. In addition, evaluation in hamsters provides insights in vaccine immunogenicity and efficacy against RSV challenge virus replication. In the present and previous studies, we found that the number of CPD ORFs correlates positively with the level of attenuation *in vitro*, in rodents and non-human primates. The stabilized CPD RSV vaccine candidate currently evaluated in children (i.e, Min L NPM2-1[N88K]; NCT04919109) was attenuated in mice, hamsters (confirming the suitability of the hamster model in predicting attenuation) [36], and non-human primates [37]. Sequential clinical evaluation, i.e., a Phase 1 evaluation of safety and immunogenicity in adults, RSV-seropositive and -seronegative children, will reveal if the level of attenuation of this vaccine candidate is appropriate. Should the vaccine candidate be over-or under-attenuated for the target population, i.e., RSV-seronegative infants and children, we will be able to fine-tune and optimize this candidate based on the restriction levels of different versions of CPD RSV in hamsters and nonhuman primates.

In conclusion, we generated a set of new CPD RSV vaccine candidates, with a range of attenuation in the hamster model, that are genetically stable and highly immunogenic and warrant further evaluation.

## Materials and methods

### Ethics statement

The hamster experiment was approved by the National Institutes of Health Institutional Animal Care and Use Committee (IACUC).

### Cell lines

African green monkey kidney (Vero) cells were grown in OptiMEM (Gibco-Life Technologies) with 5% fetal bovine serum (FBS, Hyclone) and 1% L-glutamine (Gibco-Life Technologies). Vero cells were maintained in 2% FBS during experiments. Baby hamster kidney cells that constitutively express the T7 polymerase (BSR T7/5) [43] were grown in GMEM media (Gibco-Life Technologies) with 10% FBS and 2% non-essential amino acids (Gibco-Life Technologies). Every other cell passage, 2% of gentamicin (Quality Biological) was added to the media to maintain selection for the T7-polymerase-expressing cells. Both cell lines were maintained at 37°C with 5% CO_2_.

### Viruses

Min A, Min L and Min FLC viruses were described previously [33]. The parent virus of the CPD RSVs is the recombinant virus RSV D46/6120. RSV D46/6120 was derived from the original recombinant version of wt RSV called D46, which has a 15,223-nucleotide genome (Genbank accession number KT992094). RSV D46/6120 has an identical sequence except that it contains a 112-nucleotide deletion in the 3’ non-translated region of the SH gene and five silent nucleotide point mutations in the last three codons and termination codon of the SH ORF. These mutations were previously shown to stabilize the RSV cDNA during propagation in *E*. *coli* without any effects on the replication *in vitro* and in mice [44]. The sequence numbering in the present study is based on KT992094.

### Construction and rescue of Min AL and derivatives

The Min AL antigenomic cDNA was generated by replacing the wt L ORF in Min A by the CPD L ORF from Min L using BamHI and KasI restriction sites. Mutations of interest identified during serial passage of Min AL were introduced into the Min AL antigenomic cDNA using the QuikChange Lightning Site-Directed Mutagenesis Kit (Agilent Technologies) following the manufacturer’s recommendations. Min AL and derivatives were recovered by reverse genetics as previously described [33]. A second passage of the rescued viruses was performed on Vero cells to generate working P2 virus stocks. The consensus sequence of these virus stocks was confirmed by Sanger or Illumina deep sequencing using overlapping reverse transcribed PCR amplicons generated using previously described primers [33,35].

### Virus titration by immunoplaque assay

As previously described [33], duplicate wells of Vero cells in 24-well plates were infected with 10-fold serial dilutions of virus for two h at 32°C. After seven to 10 days incubation at 32°C, cells were fixed with 80% cold methanol and immunostained using a mixture of three RSV F-specific monoclonal antibodies (MAbs) [28], and further incubated with a polyclonal goat anti-mouse-IgG(H+L) antibody conjugated to horseradish peroxidase (KPL, 5220–0341) to vizualise the plaques. Virus titers are expressed in pfu/ml.

### Multi-cycle growth kinetics

Two sets of subconfluent wells of Vero cells in six-well plates were inoculated each in duplicate with the indicated viruses using an MOI of 0.01 pfu/cell. One set of plates was incubated at 32°C for 12 days while the other set was incubated at 37°C for 10 days.Virus was harvested on the indicated days pi by scraping and harvesting the cells with the media. The cell and media suspensions were vortexed three times for 10 sec each and the supernatant was clarified by centrifugation at 1,200 rpm for 5 min at 4°C. Aliquots of viruses were snap frozen in dry ice and stored at -80°C before titration by plaque assay.

### Characterization of plaque size

As previously described [35], Vero cell monolayers in six-well plates were inoculated with 250 pfu per well of virus and incubated at 32°C for seven days with an overlay containing 0.8% methylcellulose. On day 7, plates were fixed with cold 80% methanol and incubated with a cocktail of three anti-RSV F MAbs [28] in Odyssey Blocking Buffer in PBS (Li-Cor), washed, and incubated with an R-phycoerythrin goat anti-mouse IgG(H+L) secondary antibody (Thermofisher, P-852). Plaques were visualized using the Celigo imager (Nexcelcom Bioscience) and the plaque size (μm^2^) was analyzed using Celigo software. An average of 3451 (±1200) individual plaques were analyzed per virus.

### Illumina whole-genome deep sequencing

Viral RNA was isolated from clarified supernatants collected at the end of passage 14 (stressed lineages) or 18 (control lineages) of the Min AL *in vitro* stress test using the QIAamp Viral RNA extraction kit (Qiagen). Then, the viral RNA was reverse transcribed using Superscript II Reverse Transcriptase (RT, ThermoFisher) and the cDNA was amplified by PCR using overlapping RSV-specific primers and a high-fidelity DNA polymerase (pfx DNA polymerase, Thermofisher) as described previously [36]. Overlapping PCR amplicons were purified using the QIAquick PCR Purification kit (Qiagen) and sequenced at the NIH Intramural Sequencing Center (NISC) using Illumina MiSeq. Only the sequences corresponding to the outer-most primers (nucleotides 1–23 and 15,174–15,223) were not directly determined.

Then, a reference sequence of the parental Min AL genome was prepared using the Bowtie 2 software [45]. Sequencing reads of Min AL and Min AL lineages were received as fastq files from NISC and were aligned to the Min AL reference sequence using Bowtie 2 with the very sensitive local parameter. Aligned reads were next converted from SAM to BAM files and then sorted based on genome position using SAMtools [46]. Sequencing reads were then sorted by their position on the flow cell and PCR duplicates were marked and removed using Picard Tools (https://www.broadinstitute.org/files/shared/mpg/plathumgen/plathumgen_fennell.pdf) with a maximum optical duplicate pixel distance of 2,500. After sorting reads and removing duplicates, the depth of coverage was measured with SAMtools (average coverage per lineage was 7,530 ±775 reads/nt) and single nucleotide variants were detected using LoFreq [47] with the default parameters. Analysis focused on prominent mutations, defined as detected in ≥30% of the sequencing reads.

### Single-cycle infections

As previously described [35,36], replicate monolayers of Vero cells in six-well plates were infected using an MOI of 3 pfu/cell at 32°C or 37°C with the indicated viruses. Two h after inoculation, the monolayers were washed twice with PBS and replenished with fresh media. At 24 and 48 hpi, four wells per virus were harvested: (i) one well was processed for analysis of cell-associated viral RNA by strand-specific RT-qPCR, (ii) one well was harvested for analysis of viral protein expression by flow cytometry, (iii) one well was harvested for Western blot analysis, and (iv) the last well was harvested to quantify virus titer by plaque assay as described above for the multi-cycle replication experiments.

### Strand-specific RT-qPCR

The cell-associated RNA from infected Vero cells from single-cycle infections was extracted using the RNeasy mini kit (Qiagen) following the manufacturer’s recommendations. Then, RNA was subjected to strand-specific RT-qPCR to quantify viral positive-sense (mRNA and antigenome) RNA and negative-sense genomic RNA, as described previously [33,35] with minor modifications. Briefly, five hundred ng of DNAse-treated RNA were reverse transcribed using SuperScript III First-Strand Synthesis System (Thermofisher) and a primer specific either to antigenomic/mRNA or genomic RNA. Each RT primer was linked to an unrelated oligonucleotide tag [36] to provide for specific amplification of the RT product at the following PCR step. Then, the cDNA was amplified by qPCR in triplicate with a primer corresponding to the oligonucleotide tag, a gene-specific reverse primer, and a probe. QPCR data were analyzed using the comparative threshold cycle (ΔCt) method, normalized to 18S rRNA internal control that had also been subjected to RT-qPCR in parallel using random first-strand primers and a 18S rRNA Taqman assay (Thermofisher). Data was analyzed using the relative quantification software on the Thermofisher Connect platform and expressed as log_10_ fold increase over the Min AL 24 h time point.

### Flow cytometry

As described previously [35], Vero cells from single-cycle infections (MOI of 3 pfu/cell, 32°C or 37°C) were harvested at 48 hpi, stained using the Live/Dead Fixable Near-IR or Blue Dead Cell dye (Thermofisher), washed with PBS, and fixed and permeabilized using BD Cytofix/Cytoperm (BD Biosciences). Then, cells were stained for 20 min at RT in Perm/Wash buffer (BD Biosciences) containing a cocktail of pre-titrated anti-RSV antibodies; namely an anti-RSV P monoclonal antibody (Abcam, ab94965) labeled in house with fluorescein isothiocyanate (FITC), an allophycocyanin (APC)-labeled anti-RSV N monoclonal antibody (Imgenex, NB100-64752APC), an anti-RSV G antibody (Abcam, ab94966) labeled with brilliant violet 421 (BV421, Biolegend), and a Biotin-labeled anti-RSV F monoclonal antibody (Millipore, MAB8262B-5). After incubation, cells were washed three times with Perm/Wash Buffer and incubated with a streptavidin-PE-labeled secondary antibody (BD pharmingen, 554061) for 20 min at room temperature. The cells were washed three times with Perm/Wash Buffer and resuspended in PBS. Data were acquired using a BD Symphony flow cytometer (BD Biosciences) and analyzed using FlowJo 10.8. The expression of the virus proteins N, P, G and F was analyzed on single live cells.

### Western blot analysis

Infected Vero cells from single-cycle infections were harvested in 1X NuPage LDS sample buffer (Thermofisher) diluted in PBS containing protease inhibitor (Roche). Cell lysates were homogenized using a QIAshredder spin column (Qiagen) and protein concentrations were determined by BCA assay (Pierce BCA Protein Assay kit). Thirty μg of proteins was denatured in a final composition of 1X NuPAGE LDS Sample Buffer (ThermoFisher) and 1X NuPAGE Sample Reducing Agent (Invitrogen) by heating at 90°C for 10 min before being subjected to electrophoresis on NuPAGE 4–15% Mini-PROTEAN TGX Gels (Bio-Rad) with 1X TGS Running Buffer (Bio-Rad). Odyssey Protein Molecular Weight Marker (Li-Cor) was run in parallel. Proteins were transferred to PVDF membranes using the iBlot 2 Gel Transfer Device (ThermoFisher) and stained with a primary mouse anti-RSV F (abcam, ab43812), a mouse anti-RSV P (abcam, ab94965) or a mouse anti-RSV G (abcam, 94966) antibody. The rabbit anti-Tubulin (abcam, ab52866n) antibody was used as a loading control. The secondary antibodies used were goat anti-rabbit IgG IRDye 680RD (at 1:15,000, Li-Cor, 926–68071), and goat anti-mouse IgG IRDye 800CW (1:10,000, Li-Cor, 926–32210). Membranes were scanned using Odyssey software, version 3.0 (Li-Cor).

### Hamster experiment

On day -2, serum was collected from 104 five-to-six-week old male golden Syrian hamsters (*Mesocricetus auratus*) (Envigo). At day 0, six groups of 16 hamsters were inoculated IN under isoflurane anesthesia with 6 log_10_ pfu of wt RSV, Min AL, or the indicated Min AL-derivatives. A last group of eight hamsters were left uninfected as control.

At 3 days pi, which corresponds to the peak of replication of wt RSV in hamsters, eight hamsters from each inoculated group were euthanized by carbon dioxide inhalation. Nasal turbinates (NT) and lungs were harvested and homogenized separately in Leibovitz (L-15) medium, and the suspensions were clarified by low-speed centrifugation and aliquots were flash-frozen and stored at -80°. Virus titers were determined later in duplicate by immunoplaque assay on Vero cells at 32°C using a methylcellulose layer contaning 1% Amphotericin B, 0.1% Gentamicin, and 0.06 mg/mL clindamycin phosphate. The limit of virus detection was 50 pfu/g in both the NT and lungs.

At 28 dpi, sera were isolated from the blood of all remaining hamsters to evaluate the RSV-specific antibody response. All sera were heated at 56°C for 30 min to inactivate complement prior to serology assays. The 60% plaque reduction neutralizing antibody titer (PRNT_60_) was evaluated using a complement-enhanced immunoplaque assay as described previously [33].

At 32 dpi, the remaining hamsters were challenged IN with 6 log_10_ pfu of wt RSV. Three days after challenge, hamsters were euthanized by carbon dioxide inhalation and NT, bronchoalveolar lavages (BAL) and lung tissues were harvested. Wt RSV virus titers in NT and lung tissues were determined in duplicate by plaque assay on Vero cells incubated at 32°C as described above. BAL samples were heated at 56°C for 30 min to inactivate complement prior to serology assays.

### Expression of inflammatory and antiviral genes in lungs of hamsters

One hundred μl of lung homogenate was mixed with 300 μl of TRIzol LS (Thermo Fisher) and total RNA was extracted using Phasemaker Tubes Complete System (Thermo Fisher) and PureLink RNA Mini Kit (Thermo Fisher). RNA from lung homogenates of two control hamsters (non-immunized and non-challenged) was derived from a previous study [40]. Then, 7 μl of the extracted RNA was reverse transcribed into cDNA using the High-Capacity RNA-to-cDNA Kit (Thermo Fisher). TaqMan assays (Thermo Fisher) specific for hamster’s (*Mesocricetus auratus*) inflammatory and antiviral genes [48–50] were performed using the TaqMan Fast Advanced Master Mix (Thermo Fisher) as previously described [40]. Hamster β-actin was used as a housekeeping gene. qPCR results were analyzed using the comparative threshold cycle (ΔΔC_T_) method, normalized to β-actin, and expressed as fold changes over the average expression of the two uninfected, unchallenged hamsters.

#### Expression and purification of the prefusion form of RSV F and ectodomain of G

The DS-CAV1 prefusion-stabilized form of the RSV F protein (pre-F, A2 strain) was expressed from a previously described cDNA [51] that was the kind gift of Drs. Peter Kwong and Barney Graham (VRC, NIAID, NIH). This molecule contained aa 1–513 inclusive of the F protein linked at the C-terminal end to the T4-phage fibritin trimerization domain (foldon) sequence for trimerization of F and a 6X His tag for purification. This plasmid was transfected into Expi293 cells using expifectamine. On day 6 post-transfection, the supernatant containing the F protein was harvested and clarified by centrifugation followed by filtration through a 0.2 μm PES membrane. Then, the supernatant was mixed with Ni-NTA Agarose resin (Thermofisher) for two to three h at 4°C with slow rotation. The mixture of cell supernatant/resin was poured onto a gravity-flow column (BioRad, cat # 7372512) and washed three times with binding buffer (50 mM Tris-Hcl, 400 mM NaCl). The pre-F protein was eluted using the binding buffer containing 300 mM imidazole (Sigma-Aldrich), concentrated using an Amicon filter, and purified by size exclusion chromatography in PBS using a Superdex 200 Increase 10/300 GL column (Cytiva). The purified pre-F protein was concentrated by centrifugation using an Amicon filter and aliquots were snap-frozen in liquid nitrogen and stored at -80°C until use.

The sequence encoding the ectodomain of RSV G (A2 strain) was codon-optimized for expression in human cells (Genscript) and cloned under the control of the CMV promoter in the pcDNA3.1(-) vector between the XbaI and KpnI sites. The construct encodes from the 5’-end to the 3’-end (i) the prolactin signal sequence, (ii) aa 65–298 (234 aa) of RSV G A2, (iii) the Avi tag sequence, (iv) the human rhinovirus 3C protease cleavage site, (v) an 8X His tag and (vi) the Strep-tag II. The resulting pcDNA3.1-RSV G A2 vector was transfected into Expi293 cells using expifectamine. On day 6 post-transfection, the media supernatant that contained the G protein was clarified by centrifugation and further clarified through a 0.2 μm PES vacuum filter. Then, the clarified supernatant was supplemented with 20 mM final Tris-HCl pH 8 and 300 mM final NaCl and mixed with Ni-NTA Agarose resin (Thermofisher) for two to three h at 4°C with slow rotation. The mixture was poured onto a gravity flow column (BioRad), washed three times with binding buffer (20 mM Tris-Hcl, 300 mM NaCl), and the G protein was eluted using binding buffer containing 75 mM imidazole. The eluted G protein was concentrated using an Amicon filter and purified by size exclusion chromatography in PBS using a Superdex 200 Increase 10/300 GL column (Cytiva). Aliquots of the purified G protein were snap-frozen in liquid nitrogen and stored at -80°C until use.

#### Dual IgG- and IgA-specific DELFIA ELISA

The levels of binding antibodies against the RSV pre F or G protein in the serum and BAL of hamsters were determined by a dual IgG- and IgA-specific dissociation-enhanced lanthanide fluorescent (DELFIA) time resolved fluorescence (TRF) immunoassay. Black 96–well assay plates (MaxiSorp, ThermoFisher, 437111) were coated with 100 μl/well of pre F or G protein (0.5 μg/ml for serum samples, 1 μg/ml for BAL samples) in carbonate coating buffer [50 mM; 3.7 g sodium bicarbonate and 0.64 g sodium carbonate (Sigma) in 1 L water] overnight at 4°C. Then, plates were washed three times in washing buffer [1xDPBS (Gibco) containing 0.1% IGEPAL CA-630, Millipore Sigma] using a BioTek 405 micro-plate washer and blocked with 250 μl of blocking buffer [1xDPBS containing 5% dry milk (W/V) (Carnation nonfat dry milk, Nestle,)] overnight at 4°C and washed once.

Sera and BAL samples were diluted 1:100 and 1:10, respectively, followed by 10 three-fold serial dilutions in a dilution plate (Costar, 3799) using sample dilution buffer (1xDPBS+5% dry milk+0.2% IGEPAL). Then, 100 μl of diluted samples was transferred from the dilution plate to the coated black plates in duplicate. After 1 h incubation at room temperature on a rotating shaker, plates were washed three times with 250 μl washing buffer, replenished with 250 μl washing buffer, and placed on a rotating shaker for another 20 min. After washing, 100 μl per well of a secondary antibody mixture [(goat anti-hamster IgG(H+L)-HRP, 1:10000 dilution, Invitrogen, PA1-29426, and rabbit anti-hamster IgA-biotin 1:1000 dilution (1:500 for BAL samples), Brookwood biomedical, Sab3002a)] diluted in dilution buffer was added per well. Then, plates were incubated for 1 h at room temperature on a rotating shaker, washed three times as described above, and 100 μl per well of streptavidin-europium (PerkinElmer, 1244–360) diluted 1:2000 in PBS+0.2% IGEPAL was added. After 1 h incubation at room temperature, plates were washed three times as above and 50 μl Pierce ECL (Thermo Fisher, 32106) per well was added. After 10 min incubation, plates were read on a Synergy neo (BioTek) plate reader for luminescence to detect IgG. After reading, plates were washed again as described above and 100 μl per well of pre-warmed enhancement solution (PerkinElmer, 4001–0010) was added and plates were further incubated for 20 min on a rocking shaker. Finally, plates were read with the Synergy neo plate reader using a program for time-resolved fluorescence with excitation wavelengths of 360/40 nm and emission filters of 620/40 nm to detect IgA.

The data were processed as follows: (i) the mean reading for each sample from duplicate wells was calculated, (ii), the mean reading from the blank samples was subtracted from the mean reading for each sample, (iv) the cut-off value was set to the blank mean plus three standard deviations of blank readings. Then, the IgG and IgA titers of each sample were determined by interpolating the sigmoid standard curve generated on Prism 9.0.

#### Statistical analysis

Distributions of the virus plaque area in Fig 1D were compared for statistical significance using the Wilcoxon rank test with continuity correction post hoc test. In Figs 6, 7 and 8, significant differences among data sets were evaluated using parametric one-way ANOVA with the Tukey post hoc tests for normally distributed data sets or the non-parametric Kruskall Wallis test with Dunns post hoc test for non-normal data sets. A log_10_ transformation was applied to data sets when necessary to obtain equal standard deviations among groups, a necessary requirement of both tests. In S2 and S3 Figs, significant differences among data sets were evaluated using the non-parametric Mann-Whitney test. Statistics were performed on the Prism version 8 GraphPad Software. Data were only considered significant at p≤0.05.

The numerical data used in all figures are included in S1 Data.

## Supporting information

S1 Data Excel spreadsheet containing, in separate sheets, the underlying numerical data and mean and standard deviations of each set of data for Figs 1B, 1C, 2A, 2B, 3B, 3C, 4, 5A, 5B, 5D, 6B, 7A, 7B, 7C, 8A, 8B, 8C, 9, S2 and S3. (XLSX)

S1 Fig Expression of RSV G in Vero cells (related to Fig 5C). Additional replicate Vero cell monolayers from the single-cycle infection experiment described in Fig 4 (MOI of 3 pfu/cell, 37°C) were harvested at 48 hpi (one well per virus per time point) for analysis of viral protein expression by Western blot. Cell lysates were prepared and analyzed by Western blotting using an anti-RSV G monoclonal antibody (RSV133) that detects the full-length as well as the truncated form of G [52]. Ladder, molecular weight marker. Note that staining of tubulin was not included to not interfere with the detection of all possible sizes of G, however the same amount of protein as in Fig 5C was used.
(TIF)

S2 Fig Expression of inflammatory cytokines in the lungs of hamsters at day 3 following IN inoculation. From the hamster experiment in which eight animals per group were sacrificed on day 3 post-inoculation and lung homogenates prepared, seven aliquots of clarified lung supernatants were chosen at random from the Min AL and wt RSV groups and two from uninoculated hamsters derived from a previous study [40] and processed to purify total RNA. The RNAs were reverse transcribed using random primers, and expression of 13 inflammation-related-genes was evaluated by hamster-specific Taqman assays. The qPCR data were analyzed by the comparative threshold cycle (ΔΔC_T_) method, normalized to beta-actin and expressed as fold-increase over the mean expression of each evaluated gene determined from the two uninoculated control hamsters (dashed line). In each graph, the median, min, and max values, 25^th^ and 75^th^ quartile, and individual values are shown. * = p<0.05; ** = p<0.01, Mann-Whitney test).
(TIF)

S3 Fig Expression of inflammatory cytokines in the lungs of hamsters at day 3 post-challenge with wt RSV. As described in the legend of Fig 8, at 32 dpi, eight hamsters per group including the group of eight non-immunized control hamsters were challenged IN with 6 log_10_ pfu of wt RSV. At day 3 pc, animals were euthanized and bronchoalveolar lavage (BAL), NT and lung tissues were collected from each animal. After challenge with wt RSV, seven of eight non-immunized control hamsters that exhibited high levels of wt RSV challenge virus replication were selected (Fig 8C, right panel), and 7 Min AL-immunized animals were selected randomly. Lung homogenates from these animals were processed to purify total RNA, together with lung homogenates from two control hamsters of the same source and age range from a previous study [40]. RNAs were reverse transcribed using random primers, and expression of 13 inflammation-related-genes was evaluated by hamster-specific Taqman assays. The qPCR data were analyzed by the comparative threshold cycle (ΔΔC_T_) method, normalized to beta-actin and expressed as fold-increase over the mean expression of each evaluated gene determined from the two uninoculated control hamsters (dashed line). In each graph, the median, min, and max values, 25^th^ and 75^th^ quartile, and individual values are shown. ** = p<0.01; *** = p<0.001, Mann-Whitney test).
(TIF)

S1 Table Number of CpGs and UpAs in open reading frames (ORFs) of wt and CPD RSVs. (DOCX)

S2 Table Temperature sensitivity of wt RSV and the indicated CPD RSVs on Vero cells. (DOCX)
