# Supplementary figures and images for: Intranasal respiratory syncytial virus vaccine attenuated by codon-pair deoptimization of seven open reading frames is genetically stable and elicits mucosal and systemic immunity and protection against challenge virus replication in hamsters
Source: PLoS Pathog. 2024 May 13;20(5):e1012198. doi: 10.1371/journal.ppat.1012198 (PMC11115275; doi:10.1371/journal.ppat.1012198)

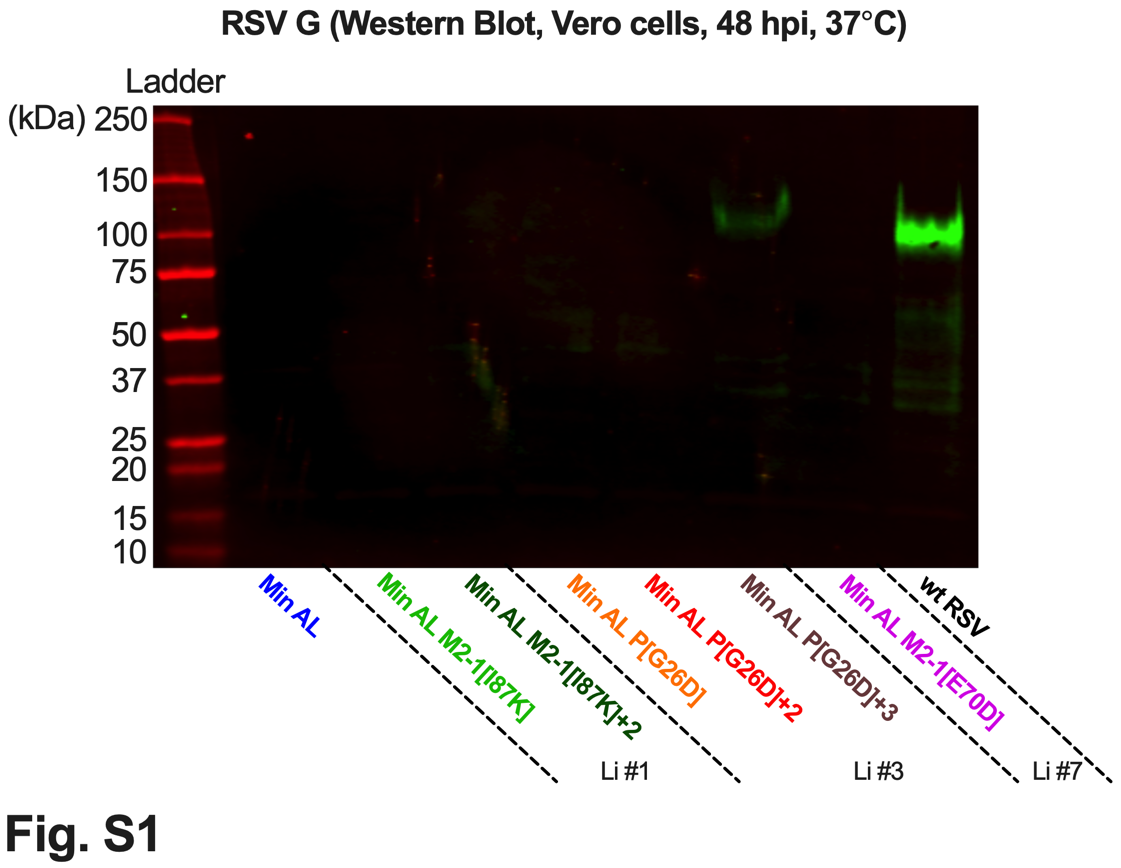

Supplement: S1 Fig — Additional replicate Vero cell monolayers from the single-cycle infection experiment described in Fig 4 (MOI of 3 pfu/cell, 37°C) were harvested at 48 hpi (one well per virus per time point) for analysis of viral protein expression by Western blot. Cell lysates were prepared and analyzed by Western blotting using an anti-RSV G monoclonal antibody (RSV133) that detects the full-length as well as the truncated form of G [52]. Ladder, molecular weight marker. Note that staining of tubulin was not included to not interfere with the detection of all possible sizes of G, however the same amount of protein as in Fig 5C was used. (TIF) [file ppat.1012198.s002.tif]

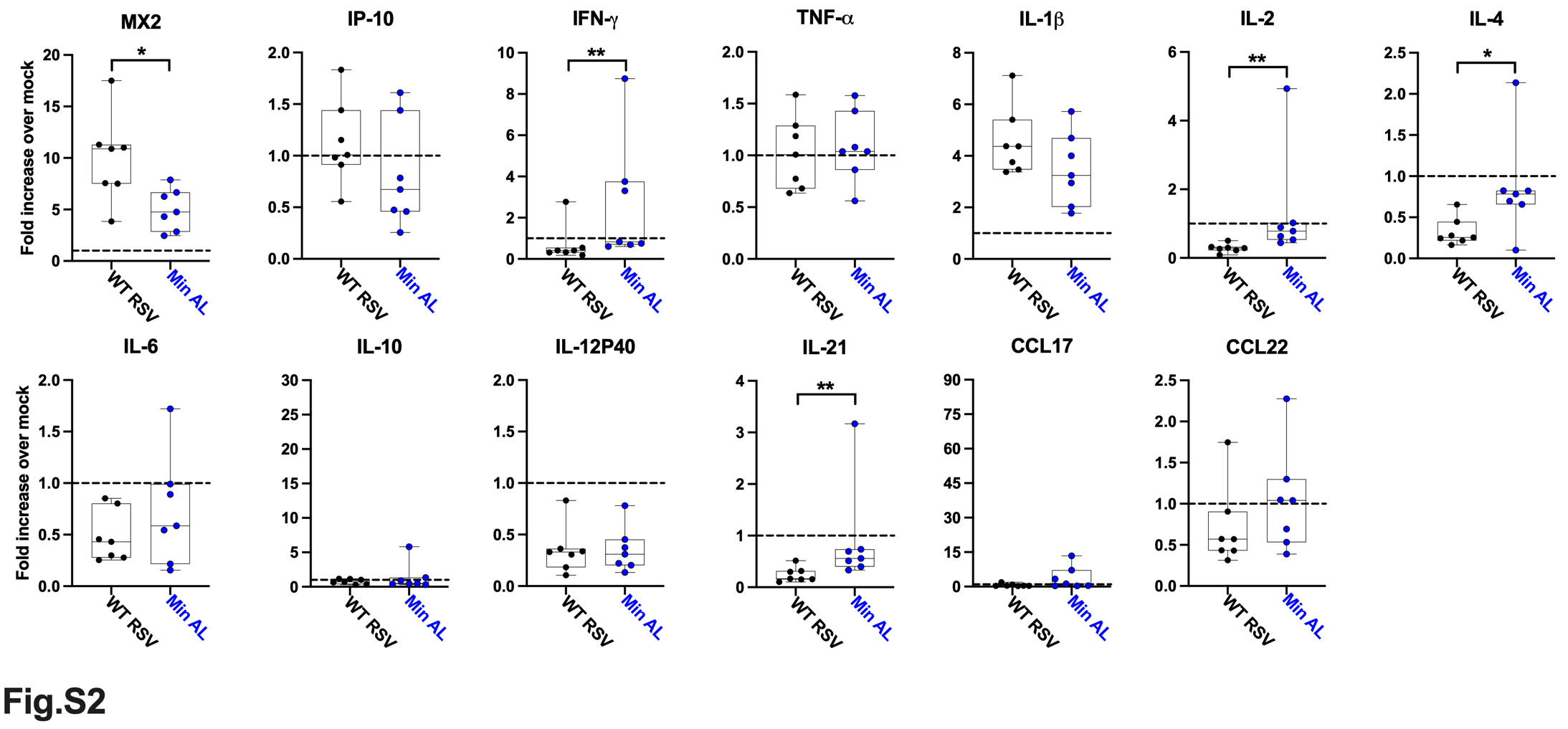

Supplement: S2 Fig — From the hamster experiment in which eight animals per group were sacrificed on day 3 post-inoculation and lung homogenates prepared, seven aliquots of clarified lung supernatants were chosen at random from the Min AL and wt RSV groups and two from uninoculated hamsters derived from a previous study [40] and processed to purify total RNA. The RNAs were reverse transcribed using random primers, and expression of 13 inflammation-related-genes was evaluated by hamster-specific Taqman assays. The qPCR data were analyzed by the comparative threshold cycle (ΔΔCT) method, normalized to beta-actin and expressed as fold-increase over the mean expression of each evaluated gene determined from the two uninoculated control hamsters (dashed line). In each graph, the median, min, and max values, 25th and 75th quartile, and individual values are shown. * = p<0.05; ** = p<0.01, Mann-Whitney test). (TIF) [file ppat.1012198.s003.tif]

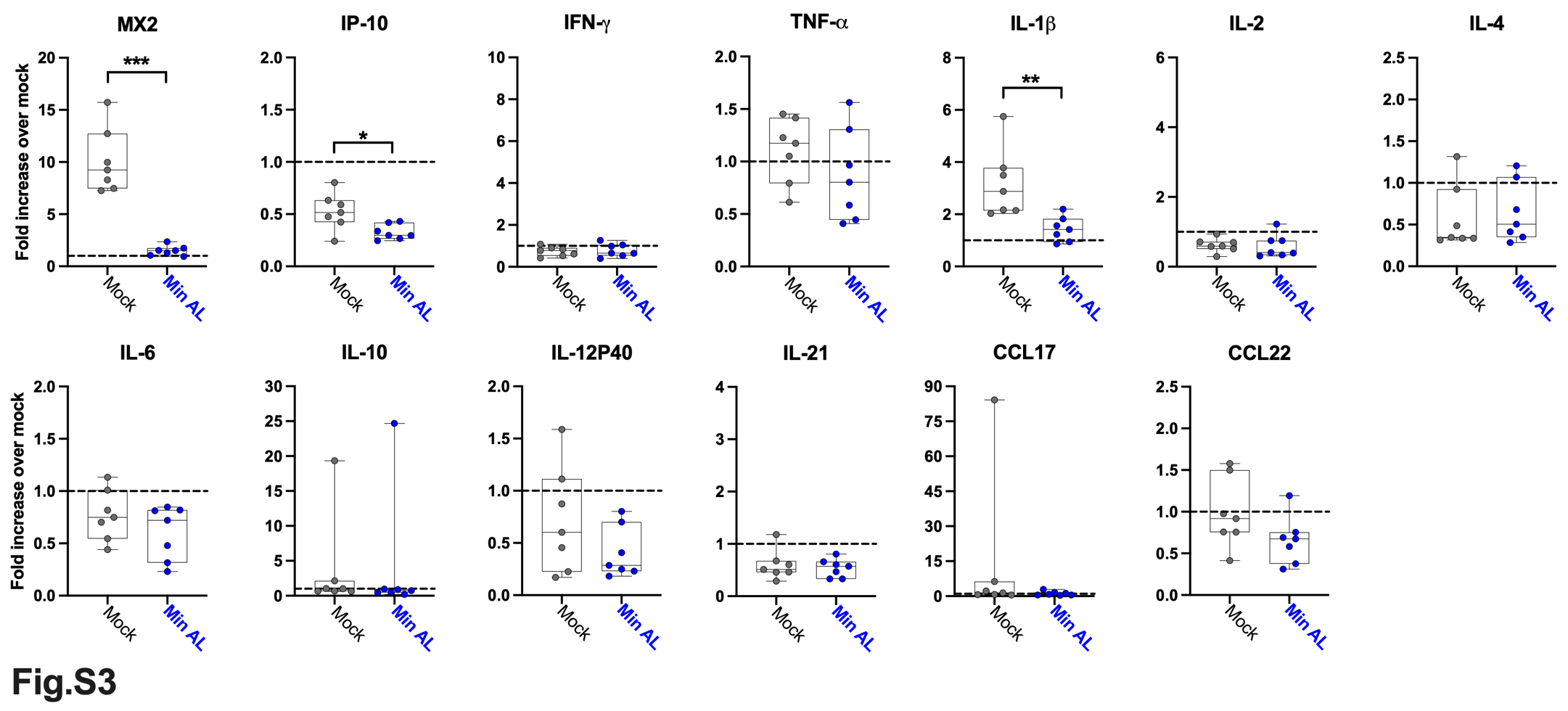

Supplement: S3 Fig — As described in the legend of Fig 8, at 32 dpi, eight hamsters per group including the group of eight non-immunized control hamsters were challenged IN with 6 log10 pfu of wt RSV. At day 3 pc, animals were euthanized and bronchoalveolar lavage (BAL), NT and lung tissues were collected from each animal. After challenge with wt RSV, seven of eight non-immunized control hamsters that exhibited high levels of wt RSV challenge virus replication were selected (Fig 8C, right panel), and 7 Min AL-immunized animals were selected randomly. Lung homogenates from these animals were processed to purify total RNA, together with lung homogenates from two control hamsters of the same source and age range from a previous study [40]. RNAs were reverse transcribed using random primers, and expression of 13 inflammation-related-genes was evaluated by hamster-specific Taqman assays. The qPCR data were analyzed by the comparative threshold cycle (ΔΔCT) method, normalized to beta-actin and expressed as fold-increase over the mean expression of each evaluated gene determined from the two uninoculated control hamsters (dashed line). In each graph, the median, min, and max values, 25th and 75th quartile, and individual values are shown. ** = p<0.01; *** = p<0.001, Mann-Whitney test). (TIF) [file ppat.1012198.s004.tif]
