# Supplementary material for: Intranasal respiratory syncytial virus vaccine attenuated by codon-pair deoptimization of seven open reading frames is genetically stable and elicits mucosal and systemic immunity and protection against challenge virus replication in hamsters
Source: PLoS Pathog. 2024 May 13;20(5):e1012198. doi: 10.1371/journal.ppat.1012198 (PMC11115275; doi:10.1371/journal.ppat.1012198)
Supplement: S1 Table — (DOCX) [file ppat.1012198.s005.docx]

**Table S1: Number of CpGs and UpAs in open reading frames (ORFs) of wt and CPD RSVs.**

|  | **Number in ORFs** | |
| --- | --- | --- |
| **Virus** | **CpG** | **UpA** |
| wt RSV | 79 | 1,312 |
| Min A | 205 | 1,418 |
| Min L | 292 | 1,514 |
| Min AL | 418 | 1,620 |
| Min FLC | 519 | 1,709 |
|  |  | |
