# Supplementary material for: Intranasal respiratory syncytial virus vaccine attenuated by codon-pair deoptimization of seven open reading frames is genetically stable and elicits mucosal and systemic immunity and protection against challenge virus replication in hamsters
Source: PLoS Pathog. 2024 May 13;20(5):e1012198. doi: 10.1371/journal.ppat.1012198 (PMC11115275; doi:10.1371/journal.ppat.1012198)
Supplement: S2 Table — (DOCX) [file ppat.1012198.s006.docx]

**Table S2: Temperature sensitivity of wt RSV and the indicated CPD RSVs on Vero cells.**

|  |  | Virus titer (log_10_ PFU per ml) at indicated temperature (°C) ^a^ | | | | | | | | | |  |  |
| --- | --- | --- | --- | --- | --- | --- | --- | --- | --- | --- | --- | --- | --- |
| **Virus** | **32** | | **35** | **36** | **37** | **38** | **39** | | **40** | | **T_SH_** | |  |
| Min A | 6.9 | | 6.6 | 6.2 | 5.7 | 5.4 | 5.3 | | **3.7** | | **40** | |  |
| Min L | 7.4 | | 7.0 | 6.6 | 5.6 | **4.2** | >1 | | >1 | | **38** | |  |
| Min AL | 7.2 | | 6.3 | **4.0** | 3.5 | >1 | >1 | | >1 | | **36** | |  |
| Min FLC | 6.1 | | 5.9 | **3.3** | >1 | >1 | >1 | | >1 | | **36** | |  |
| wt RSV | 7.1 | | 6.8 | 6.8 | 6.8 | 6.7 | 6.7 | | 6.6 | | **>40** | |  |
|  |  | | |  |  |  | |  | |  | | | |

^a^ The *ts* phenotype for each virus was evaluated by assessing virus growth on Vero at the indicated temperatures utilizing temperature controlled water-baths. For viruses with a *ts* phenotype, the shut-off temperatures (T_SH_) are underlined and listed in boldface at the right. T_SH_ is defined as the lowest restrictive temperature at which there is a reduction in plaque number compared to 32°C that is 100-fold or greater than that observed for wt RSV at the two temperatures. The *ts* phenotype is defined as having a T_SH_ of 40ºC or less.
